# A novel function for p21Cip1 and acetyltransferase p/CAF as critical transcriptional regulators of TGFβ-mediated breast cancer cell migration and invasion

**DOI:** 10.1186/bcr3322

**Published:** 2012-09-20

**Authors:** Meiou Dai, Amal A Al-Odaini, Ani Arakelian, Shafaat A Rabbani, Suhad Ali, Jean-Jacques Lebrun

**Affiliations:** 1 Division of Medical Oncology, Department of Medicine, McGill University Health Center, Royal Victoria Hospital, Montreal, QC, Canada; 2 Department of Medicine, McGill University Health Center, Royal Victoria Hospital, Montreal, QC, Canada

## Abstract

**Introduction:**

Tumor cell migration and invasion are critical initiation steps in the process of breast cancer metastasis, the primary cause of breast cancer morbidity and death. Here we investigated the role of p21Cip1 (p21), a member of the core cell cycle machinery, in transforming growth factor-beta (TGFβ)-mediated breast cancer cell migration and invasion.

**Methods:**

A mammary fat pad xenograft mouse model was used to assess the mammary tumor growth and local invasion. The triple negative human breast cancer cell lines MDA-MB231 and its sub-progenies SCP2 and SCP25, SUM159PT, SUM149PT, SUM229PE and SUM1315MO_2 _were treated with 5 ng/ml TGFβ and the protein expression levels were measured by Western blot. Cell migration and invasion were examined using the scratch/wound healing and Transwell assay. TGFβ transcriptional activity was measured by a TGFβ/Smad reporter construct (CAGA12-luc) using luciferase assay. q-PCR was used for assessing TGFβ downstream target genes. The interactions among p21, p/CAF and Smad3 were performed by co-immunoprecipitation. In addition, Smad3 on DNA binding ability was measured by DNA immunoprecipitation using biotinylated Smad binding element DNA probes. Finally, the association among active TGFβ/Smad signaling, p21 and p/CAF with lymph node metastasis was examined by immunohistochemistry in tissue microarray containing 50 invasive ductal breast tumors, 25 of which are lymph node positive.

**Results:**

We found p21 expression to correlate with poor overall and distant metastasis free survival in breast cancer patients. Furthermore, using xenograft animal models and *in vitro *studies, we found p21 to be essential for tumor cell invasion. The invasive effects of p21 were found to correlate with Smad3, and p/CAF interaction downstream of TGFβ. p21 and p/CAF regulates TGFβ-mediated transcription of pro-metastatic genes by controlling Smad3 acetylation, DNA binding and transcriptional activity. In addition, we found that active TGFβ/Smad signaling correlates with high p21 and p/CAF expression levels and lymph node involvement using tissue microarrays from breast cancer patients.

**Conclusions:**

Together these results highlight an important role for p21 and p/CAF in promoting breast cancer cell migration and invasion at the transcriptional level and may open new avenues for breast cancer therapy.

## Introduction

p21 was originally identified as a cell cycle regulator through inhibition of different cyclin/cyclin-dependent kinase complexes [1]. p21 is a member of the Cip/Kip family of cell cycle inhibitors, which also includes p27Kip1 and p57Kip2 [2-4]. In addition to its role in cell cycle control, p21 is involved in the regulation of cellular senescence, gene transcription, apoptosis and actin cytoskeleton [5-7]. The role of p21 in breast cancer development and progression has not been fully investigated. While p21 is involved in cell cycle control and is a downstream target of the tumor suppressor p53, it does not fulfill the classic definition of a tumor suppressor. Germline or somatic mutations in the p21 gene are not common in human cancers [8]. Furthermore, *in vivo *studies using p21 knockout mice showed that, while loss of p21 expression efficiently blocked the ability of the cells to undergo G1 arrest following DNA damage, these animals developed normally [9]. Intriguingly, p21 is often overexpressed in aggressive tumors, including carcinomas of the pancreas, breast, prostate, ovary and cervix [10-13]. Together these observations suggest that the role played by p21 in cancer is more complex than initially thought and that, in addition to its well-known cell cycle regulatory effect, it may have uncharacterized roles in promoting carcinogenesis.

Tumor cell migration and invasion are critical steps in the metastatic process and are regulated by numerous tumor-secreted factors which modify the tumor microenvironment by acting on stromal recruitment and extracellular matrix (ECM) degradation, resulting in tumor cell migration and invasion [14]. Among these tumor-secreted factors, TGFβ has been shown to play a pivotal role in promoting tumor metastasis [15]. The TGFβ family regulates asymmetric cell division and cell fate determination during embryogenesis and exerts profound effects on reproductive functions, immune responses, cell growth, bone formation, tissue remodeling and repair throughout adult life [16]. The effects of TGFβ in breast cancer are complex. TGFβ is thought to play a dual role in breast cancer progression, acting as a tumor suppressor in normal and early carcinoma, and as a pro-metastatic factor in aggressive carcinoma [17]. The growth inhibitory effects of TGFβ are known to be mediated through transcriptional repression of the c-myc gene [18] and induction of the cell cycle inhibitors p15Ink4b (p15) and p21, leading to G1 arrest [19,20]. During tumor progression, however, the loss of TGFβ growth-inhibitory effects is frequently due to defects in c-myc and p15 regulation by TGFβ [18]. Meanwhile, other TGFβ responses prevail, unrelated to growth inhibition and favoring tumor progression and metastasis [21-25]. Indeed, TGFβ induces degradation of the ECM, inhibits cell adhesion and stimulates cell migration and invasion, thereby promoting tumor metastasis [21-23,25]. Moreover, during cancer progression, tumor cells secrete increasing quantities of TGFβ, which in turn alter the stroma environment, leading to stimulation of tumor angiogenesis and causing local and systemic immunosuppression, thus further contributing to tumor progression and metastasis [21-23,25]. Together these studies highlight an important role for TGFβ in advanced breast cancer. However, the function for p21 downstream of TGFβ has not been described in breast cancer.

In this study, we found that high p21 expression correlates with poor survival in breast cancer patients. The expression of p21 is required to promote tumor cell migration and invasion *in vitro *and local invasion *in vivo*. Furthermore, p21 expression is tightly regulated by TGFβ/Smad3 signaling in a panel of human basal-like triple negative breast cancer cell lines. We found p21 to physically interact with Smad3 and the histone acetyltransferase p/CAF in response to TGFβ and identified p21 and p/CAF as key regulators of TGFβ-mediated breast cancer cell migration and invasion. We also showed that p21 and p/CAF regulate TGFβ transcriptional activity on multiple tumor-promoting target genes by controlling Smad3 acetylation and Smad3 occupancy on its DNA binding elements. Immunohistochemical analysis of tissue arrays from breast cancer patients revealed a significant correlation between active TGFβ/Smad3 signaling and high expression levels of both p21 and p/CAF in lymph node-positive invasive ductal carcinomas. Together, our findings identified p21 and p/CAF as critical regulators of cell migration and invasion downstream of TGFβ/Smad3 pathway in advanced breast cancer.

## Methods

### Cell culture and transfection

Human breast carcinoma MDA-MB231, SCP2 and SCP25 cells (provided by Dr. Joan Massagué) and HEK293 cells were grown in DMEM supplemented with 10% fetal bovine serum (FBS) and 2 mM L-glutamine at 37°C in 5% CO_2_. SUM149PT, SUM159PT and SUM229PE (provided by Dr. Stephen P Ethier) were grown in F-12 HAM'S nutrient mixture (HyClone Laboratories, Inc. Logan, Utah, USA) supplemented with 5% FBS, 5 µg/ml insulin (Sigma-Aldrich, St. Louis, MO, USA), 1 µg/ml hydrocortisone (Sigma) at 37°C in 5% CO_2_. SUM1315MO_2 _were grown in F-12 HAM'S nutrient mixture (HyClone) supplemented with 5% FBS, 5 µg/ml insulin (Sigma), 10 ng/ml epidermal growth factor (EGF) (Sigma) at 37°C in 5% CO_2_.

Cells were transfected with different p21, p/CAF, Smad2 and Smad3 siRNAs (Sigma), 6× myc- Smad2, myc-Smad3, p/CAF (Addgene plasmid 8941) [26] and Flag-tagged human p21 cDNAs (Addgene plasmid 16240) [27] using Lipofectamine™ 2000 reagent (Invitrogen, Carlsbad, CA, USA ), according to the manufacturer's protocol. MDA and SCPs cells were serum-starved for 24 hrs and stimulated or not with 5 ng/ml TGFβ1 (PeproTech, Rocky Hill, NJ, USA) in DMEM supplemented with 2 mM L-glutamine. For stable cell line generation, SCP2 cells were transfected with p21 shRNA (Santa Cruz Biotechnology, Santa Cruz, CA, USA) and pools of stable cells were selected with 10 ng/ml puromycin (Invitrogen). SUM159PT cells were serum-starved for 24 hrs in the absence of insulin and hydrocortisone before TGFβ1 stimulation.

### Western blot analysis and immunoprecipitation

Cells were lysed in cold extraction buffer (10 mM Tris-HCl, pH 7.5, 5 mM EDTA, 150 mM NaCl, 30 mM sodium pyrophosphate, 50 mM sodium fluoride, 1 mM sodium orthovanadate, 1% Triton X-100) containing protease inhibitors (1 mM phenylmethylsulfonyl fluoride, 10 µg/ml leupeptin hydrochloride, 10 µg/ml aprotinin and 10 µg/ml pepstatin A). The lysates were then centrifuged at 14,000 rpm for 15 minutes at 4°C. Protein content was measured using BCA protein assay kit (Thermo Scientific, Rockford, IL, USA). Equal protein was analyzed by Western blot using mouse anti-p21 (F5), mouse anti-c-myc, mouse anti-p15, rabbit anti-Smad2/3 (1:1,000 dilution, Santa Cruz Biotechnology), phospho-cofilin and cofilin antibodies (1:1,000 dilution, Millipore, Billerica, MA, USA), and followed by secondary antibodies goat anti-mouse or rabbit. Immunoprecipitations were performed overnight at 4°C using antibodies against p300/CBP (Santa Cruz Biotechnology), p/CAF (Abcam^®^, Cambridge, MA, USA) and p21. Protein G-Sepharose (GE Healthcare Bio-Sciences, Piscataway, NJ, USA) was added for 1 hr at 4°C, and washed four times with cold lysis buffer. The immunocomplexes were boiled with 2× sodium dodecyl sulfate (SDS) Laemmli sample buffer for five minutes and subjected to immunoblotting.

### Histone proteins extraction

Total histone proteins were extracted as previously described [28]. Briefly, 80% confluent of SCP2 cells from a 100-mm tissue culture plate were serum-starved for 24 hrs and stimulated with or without 5 ng/ml TGFβ or 1 µM trichostatin A (TSA). SCP2 cells were harvested and resuspended in cold hypotonic lysis buffer containing 10 mM Tris-HCl, pH 8.0, 1 mM KCl, 1.5 mM MgCl_2_, 1 mM DTT, protease inhibitors, 1 µM TSA and 10 mM sodium butyrate. Cell lysates were rotated at 4°C for 30 minutes and then centrifuged at 10,000 g, 4°C, for 10 minutes. The supernatants were discarded and nuclei pellets were resuspended in 400 µl of 0.4 N H_2_SO_4 _and incubated overnight on a rotator at 4°C. Samples were centrifuged at 16,000 g for 10 minutes and supernatants containing histones were transferred into a fresh tube. A total of 132 µl trichloroacetic acid was added drop by drop to the histone solution, inverted several times and then incubated on ice for 30 minutes. The histone precipitates were centrifuged at 16,000 g for 10 minutes and pellets were washed twice with ice-cold acetone and the histone pellets were air dried for 20 minutes. Total histone proteins were subjected to Western blot analysis using an acetylated lysine antibody (Millipore).

### DNA affinity precipitation assay

SCP2 cells transiently transfected with the indicated siRNAs were stimulated with TGFβ for 30 minutes. Cell lysate were extracted in cold lysis buffer containing 10 mM Tris-HCl, pH 7.4, 150 mM NaCl, 1 mM EDTA, 1% Nonidet P-40, 30 mM sodium pyrophosphate, 1 mM sodium orthovanadate and protease inhibitors as described above. A total of 5 µg Poly (dI-dC) competitor (Sigma) was incubated with 1 mg of total cell lysate for 30 minutes at 4°C. A total of 500 pmol of double-stranded oligonucleotides (IDT) was added and incubated with cell lysates for two hours at 4°C. Streptavidin-agarose beads (65 µl; Sigma) were added, incubated overnight at 4°C and then washed three times with cold lysis buffer. The streptavidin-agarose beads containing biotinylated oligonucleotides and protein complex were boiled with 2× SDS Laemmli sample buffer for five minutes and subjected to immunoblotting. The sequences of biotin labeled double-strand oligonucleotides (IDT) were previously described [29]. For Smad binding element oligonucleotide (4× SBE), Sense: biotin-5'-CAGACAGTCAGACAGTCAGACAGTCAGACAGT-3', antisense: 5'-ACTGTCTGACTGTCTGACTGTCTGACTGTCTG-3'. For control oligonucleotide, sense: biotin-5'-GCCCAGGCGCACCTGCTCCGATATCAATATCCGGC-3', anti-sense, 5'- GCCGGATATTGATATCGGAGCAGGTGCGCCTGGGC-3'.

### Luciferase assays

SCP2 cells were transiently co-transfected with 50 nM Scr siRNA, 50 nM p21 siRNA or 0.5 µg flag-tagged p21 cDNA in combination with 0.3 µg SBE reporter construct (CAGA12-luc) and 0.1 µg pCMV-β-gal. Transfected cells were then stimulated with or without 5 ng/ml TGFβ for 16 hrs. Luciferase activity of CAGA12-luc was measured (EG & G Berthold luminometer, Berthold Technologies, Bad Wildbad, Baden-Württemberg, Germany) and normalized to β-galactosidase activity.

### Real-Time PCR

Total RNA was extracted using TRIzol reagents (Invitrogen). Reverse transcription of total RNA using random primers was carried out using M-MLV reverse transcriptase (Invitrogen) as per the manufacturer's instructions. Real-time PCRs were carried out using SsoFast™EvaGreen^® ^Supermix (Bio-Rad, Hercules, CA, USA) in a Rotor Gene 6000 PCR detection system (MBI Lab Equipment, Montreal Biotech Inc. Kirkland, PQ, Canada). PCR conditions were as follows: 95°C for 30 s, 40 cycles (95°C for 5 s and 60°C for 20 s). The primer sequences were as follows: *IL8 *forward primer, GCAGAGGCCACCTGGATTGTGC; reverse primer, TGGCATGTTGCAGGCTCCTCAGAA; *IL6 *forward primer, CTCCCCTCCAGGAGCCCAGC; reverse primer, GCAGGGAAGGCAGCAGGCAA; *PLAU *forward primer, GCCCTGGTTTGCGGCCATCT; reverse primer, CGCACACCTGCCCTCCTTGG; *MMP9 *forward primer, TGGACACGCACGACGTCTTCC; reverse primer, TAGGTCACGTAGCCCACTTGGTCC; *PTGS2 *forward primer, AGCTTTCACCAACGGGCTGGG; reverse primer, AAGACCTCCTGCCCCACAGCAA; *TGFBI *forward primer, CGGCTGCTGCTGAAAGCCGACCA; reverse primer, GGTCGGGGCCAAAAGCGTGT; p21 forward primer, TGTCCGCGAGGATGCGTGTTC; reverse primer, GCAGCCCGCCATTAGCGCAT; *GAPDH *forward primer, GCCTCAAGATCATCAGCAATGCCT; reverse primer, TGTGGTCATGAGTCCTTCCACGAT.

### Thiazolyl blue tetrazolium bromide (MTT) assay

A total of 100 µl of cell suspension (1× 10^5 ^cells/ml in DMEM supplemented with 1% FBS) was stimulated or not in the presence or absence of 5 ng/ml TGFβ and cultured in 96-well plates for two days. After two days, 25 µl 5 mg/ml MTT solution (Sigma) was added to each well and incubated for two hours. A total of 200 µl of dimethyl sulfoxide (DMSO) was added to each well and mixed well. The absorbance at 570 nm was measured on a plate reader.

### Cell cycle analysis

SCP2 cells were stimulated with TGFβ for 0, 2, 6 and 24 hrs. Cells were then fixed with 70% ethanol overnight, treated with 20 µg/ml RNase (Sigma), and stained with 0.5 mg/ml propidium iodide (Santa Cruz Biotechnology). DNA content was determined using a FACScan flow cytometry analyzer.

### Kinetic cell migration assay

Cells were transfected with different siRNAs and plated in Essen ImageLock 96-well plates (Essen Bioscience, Ann Arbor, Michigan, USA) at 50,000 cells per well. The use of ImageLock 96-well plates ensures that images/videos of the wound are automatically taken at the exact same location by the IncuCyte™software (Essen Bioscience). Cells were then serum-starved for six hours and confluent cell layers were scratched using the Essen Wound maker to generate approximately 800 µm width wounds. After wounding, cells were washed two times with PBS and stimulated in the presence or the absence of 5 ng/ml of TGFβ. ImageLock 96-well plates were then placed into IncuCyte (Essen Bioscience) and imaged every hour for 24 hrs. The data were analyzed by three integrated metrics: wound width, wound confluence or relative wound density automatically measured by the IncuCyte software.

### Matrigel invasion assay

For the Transwell assays, 30 µl of growth factor reduced (GFR) Matrigel (BD Biosciences, diluted 1:3 in pre-chilled H_2_O) was coated onto each insert of 24-Tranwell invasion plate (8-µm pore size; BD Biosciences) and incubated for two hours in the cell culture incubator. SCP2 or SUM159PT (6 × 10^4 ^cells/insert) were seeded on Transwell Insert coated GFR-Matrigel and cells in the upper chamber were stimulated or not with 5 ng/ml TGFβ for 24 hrs. For SCP2 cells, bottom chambers contained 10% FBS in DMEM medium. For SUM159PT cells, bottom chambers were added to F-12 HAM'S medium with 5% FBS. After 24 hrs, cells from the upper chamber were removed by cotton swab and cells invaded through GFR-Matrigel were fixed with 3.7% formaldehyde for 10 minutes and then stained with 0.2% crystal violet for 20 minutes. Images of the invading cells were photographed using an inverted 4× or 10× microscope and total cell numbers were counted and quantified by Image J software (National Institute of Health, Bethesda, Maryland, USA).

### Immunofluorescence microscopy

Cells were grown on coverslips at 50% confluence, stimulated or not with TGFβ overnight. Cells were then fixed with 3.7% formaldehyde for 10 minutes and permeabilized in 0.1% Triton X-100 for 3 minutes, washed with PBS and blocked for 1 hr in 2% BSA. Cells were then incubated with anti-p21 antibody for one hour, washed with PBS and incubated with the secondary antibody Alexa Fluor®568 goat anti-rabbit IgG (1:800 dilution; Invitrogen) for one hour. Stained coverslips were mounted with SlowFade® Gold antifade reagent with DAPI (Invitrogen). Confocal analysis was performed using a Zeiss LSM 510 Meta Axiovert confocal microscope (Carl Zeiss, Oberkochen, Baden-Württemberg, Germany) using 63× objective.

### Immunohistochemistry, scoring and statistical analysis

Tissue sections (5 µm) from breast carcinoma microarray slides (BCR961 and T088, Biomax) were deparaffinized and rehydrated. The patient characteristics are in Table S1 (Additional file 1). The slides were then placed in 10 mM citrate buffer (pH 6.0) and boiled at 95°C for 15 minutes. The primary antibodies used for immunohistochemistry staining were AE1/AE3 (Thermo Scientific), p21 (c-19, Santa Cruz Biotechnology), p/CAF (ab12188, Abcam), phospho-Smad3 (Cell Signaling). HRP Polymer & DAB Plus Chromogen (Thermo Scientific) was used for detection of p21, p/CAF and phospho-Smad3. The slides were then counter stained with hematoxylin (Vector Laboratories, Burlingame, CA, USA) and dehydrated and mounted for microscopic examination. All images were scanned by ScanScope digital scanners (Aperio, Vista, CA, USA). All samples were reviewed and scored by a pathologist. The staining for p21, p/CAF and phospho-Smad3 was scored from 0 to 4 as follows: 0, no staining; 1, <25% tumor cells stained weakly; 2, 25 to 50% tumor cells stained moderately; 3, >50% tumor cells stained moderately; 4, >50% tumor cells stained strongly.

Correlations between phospho-Smad3, p/CAF and p21 were examined by the Pearson correlation test using SPSS 19 software (IBM, Armonk, NY, USA). Associations between these protein expressions and lymph node status were assessed by Fisher's exact test. *P*-value (two-sided) <0.05 was considered statistically significant.

### Mammary fat pad and intratibia injections of nude mice

Four- to six-week old female Balb/c nude mice were obtained from Charles River (Charles River Laboratories International, Wilmington, MA, USA) and used as a model for primary mammary tumor formation and local invasion. The animal study was approved by the ethics committee and all the experimental animal protocols were in accordance with the McGill University Animal Care. Following the administration of an anesthetic cocktail of ketamine (50 mg/kg), xylazine (5 mg/kg) and acepromazine (1 mg/kg) injected intramuscularly into the mice, parental and shRNA p21 SCP2 cells were inoculated at 5× 10^5 ^cells per mouse in 100 μl of saline (20% Matrigel) with a 30-gauge needle into the mammary pad. The tumor size was measured once a week using a caliper. Tumor volume was determined according to the formula: tumor volume = shorter diameter^2 ^× longer diameter/2. Sets of mice (eight per group) were sacrificed at eight weeks post-injection to examine invasiveness of the primary tumor. At the end of these studies, mammary tumors with surrounding fat pad and tissues were fixed in 10% neutral-buffered formalin for one day. Sections of mammary tumor were embedded in Tissue-Tek O.C.T. (VWR International, Radnor, PA, USA) compound and 9 µm thick sections were stained with hematoxylin and eosin. Images of the tumors were photographed by light microscope using 10× and 20× objectives.

For intratibia injections, parental and shRNA p21 SCP2 cells (2.0× 10^6^) were injected intramuscularly into the left tibia of two group mice (eight per group). The mice were monitored weekly for tumor burden. Digital radiography of the hind limbs of all animals was used to monitor the development of skeletal lesions at four, six and eight weeks post-injection in a MX-20 cabinet X-ray system (Faxitron Bioptics, Tucson, Arizona, USA). On Week 8, radiographs of anesthetized mice were taken and the osteolytic lesion area was analyzed as previously described [30]. The score of lesion area was measured as 0, no lesions; 1, minor lesions; 2, small lesions; 3, significant lesions with minor break of margins (1% to 10% of bone surface damaged); 4, significant lesions with major break in peripheral lesions (>10% of bone surface damaged).

### Statistical analyses

Student's *t*-test was used and differences between groups were considered significant at *P < 0.05.

## Results

### p21 expression correlates with poor survival in breast cancer patients

**Figure 1:**
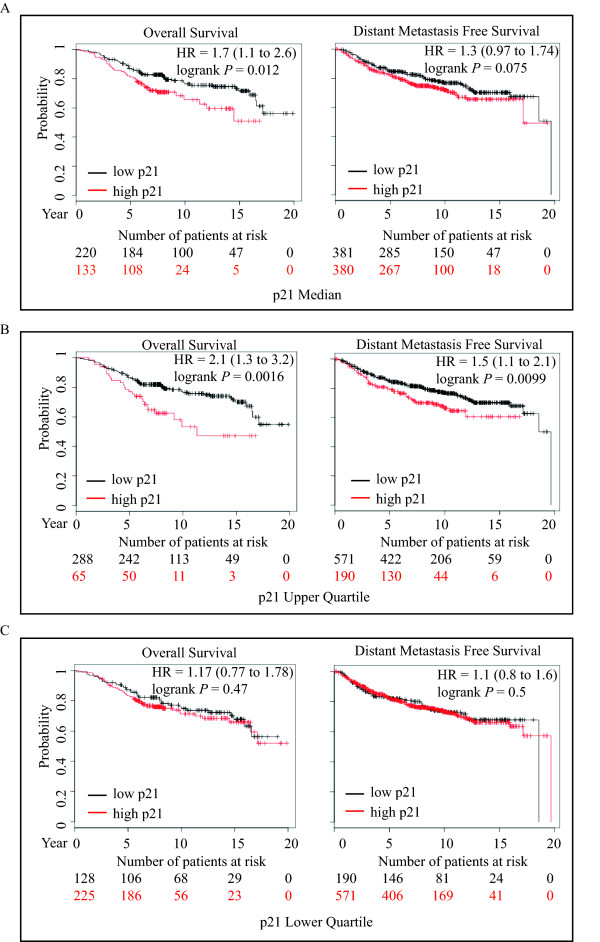
**High p21 expression correlates with poor survival in breast cancer patients**. **A-C**, The relationship of higher p21 expression and breast cancer outcome was assessed by Kaplan-Meier survival analysis. The survival rates were analyzed using three different and separate splits of the patients, based on p21 expression levels (A, median; B, upper quartile; C, lower quartile). For each split, the patients were then divided into high and low groups with respect to p21 expression. Overall survival (left panels) and distant metastasis-free survival (right panels) correlate with p21 gene expression. Number of breast cancer patients at risk with higher expression (red) and lower expression (black) of p21 at the indicated time points.

Previous studies have suggested that higher expression of cytoplasmic p21 correlated with poor prognosis in breast carcinomas [31-33]. To further explore the correlation of p21 gene expression level with clinical outcome in breast cancer patients, we utilized a recently published gene profiling database of breast cancer patients to assess p21 gene expression in overall survival (OS) and distant metastasis-free survival (DMFS) outcomes [34]. We analyzed the prognostic value according to the median, upper and lower quartile expression levels of p21 in the 20-year follow-up for OS (353 patients) and DMFS (761 patients). As shown in Figure 1A-C (left panels), elevated p21 expression significantly correlated with poor OS in both median (Hazard Ratio (HR), 1.7; 1.1 to 2.6; *P *= 0.012) and upper quartile (HR, 2.1; 1.3 to 3.2; *P *= 0.0016), but not in the lower quartile (HR, 1.17; 0.77 to 1.78; *P *= 0.47). Furthermore, higher p21 levels showed a similar pattern (HR, 1.3; 0.97 to 1.74; *P *= 0.075) in DMFS (Figure 1A-C, right panels). After 20 years follow-up, patients who are free of distant metastasis showed reduced expression of the p21 gene and a better survival rate. Although the prediction did not show statistically significant results in the median expression, the *P*-value of the p21 upper quartile (HR, 1.5; 1.1 to 2.1; *P *= 0.0099) did reach statistical significance (Figure 1B, right panel). We also analyzed the relationship of p21 expression and clinical outcomes in both estrogen receptor positive (ER+) and negative (ER-) breast cancer patients. p21 expression is highest in patients with poor prognosis regardless of ER status (Additional file 2, Figure S1). Even though one cannot rule out that elevated p21 levels could also be found in the stroma rather than the tumor cells themselves, these data demonstrate that high p21 expression correlates with poor clinical outcomes and suggest that elevated p21 expression may play a role in promoting tumor progression.

### Silencing p21 prevents breast tumor local invasion *in vivo *and cancer cell migration and invasion *in vitro *

**Figure 2:**
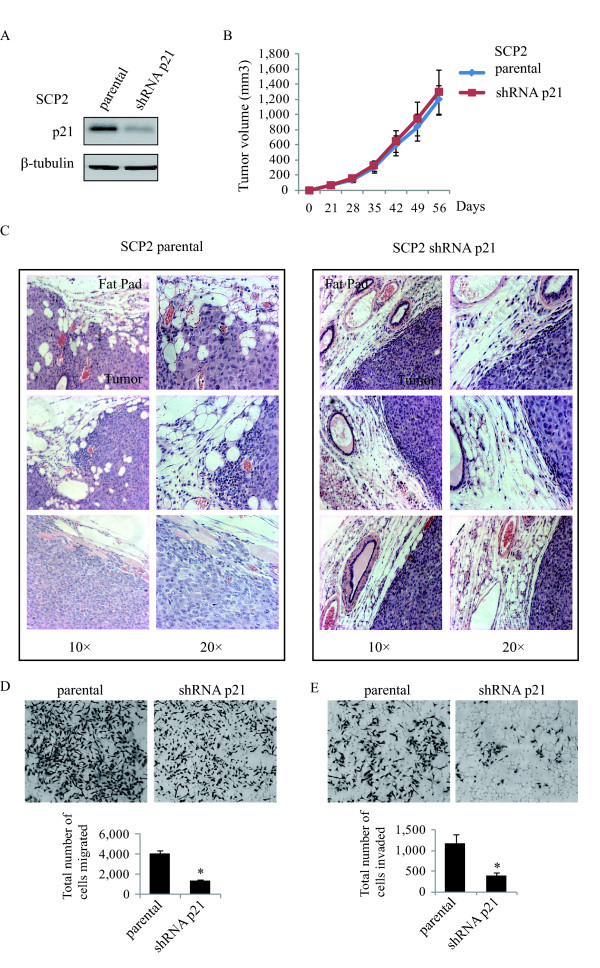
**Silencing p21 prevents breast tumor local invasion *in vivo *and cancer cell migration and invasion**. **A**, Total lysates from parental and shRNA p21 SCP2 cells were analyzed by immunoblotting for the protein levels of p21 and β-tubulin. **B**, Parental and shRNA p21 SCP2 cells were injected into the mammary glands of four- to six-week-old female Balb/c nude mice. The size of mammary tumor was measured from two sets of mice (eight per group; error bars indicate SEM). **C**, Representative photographs show hematoxylin and eosin staining of the mammary gland (tumor and fat pad) of 12- to 15-month-old mice. **D **and **E**, Transwell cell migration (left panel) and GFR-Matrigel invasion assay (right panel) of parental and shRNA p21 SCP2 cells were performed. Graphs show total migrated and invaded cell number counted by Image J (error bars indicate SEM; n = 3 independent experiments). Student's unpaired *t*-test was used to compare parental vs. shRNA p21 SCP2 groups. Differences were considered significant at *P < 0.05.

To investigate the contribution of p21 to tumor formation and progression in breast cancer, we used a bone-metastatic cell line SCP2, a sub-progeny of the human triple negative breast cancer MDA-MB231 (hereafter referred to as MDA) cells [35]. We first assessed the effect of suppressing p21 on tumor growth using a mammary fat pad xenograft mouse model. A specific p21 shRNA was stably transfected to generate a pool of p21-deficient SCP2 cells. Knockdown of p21 using shRNA efficiently reduced p21 protein expression, as compared to parental SCP2 cells (Figure 2A). Parental and shRNA p21 SCP2 cells were orthotopically injected into the mammary fat pad of female Balb/c nude mice (eight mice per group). Tumor growth was monitored weekly. There was no difference in the rate of primary tumor formation or tumor size between animals injected with parental or p21-deficient cells (Figure 2B), suggesting p21 is not likely involved in tumor formation. Next, we evaluated the effect of p21 depletion on tumor invasiveness, a critical step for early tumor progression. Intact tumors were taken with the overlaying skin and surrounding deep tissues and analyzed by a pathologist. Tumor invasiveness was assessed by determining the extent of infiltration of cancer cells to the surrounding tissue (stroma, fat pad, skin and muscle tissue), as previously described [36]. As shown in Figure 2C (left panels), tumors from the parental SCP2 group displayed no clear margin with the surrounding tissues and were deeply invading into nearby structures. In contrast, tumors derived from animals transplanted with p21-depleted SCP2 cells formed a well-encapsulated tumor mass that did not invade the surrounding tissues (Figure 2C, right panels), strongly suggesting that p21 plays an important role in tumor invasion. This was confirmed *in vitro*, as p21 gene silencing in SCP2 cells inhibited both cell migration and invasion (Figure 2D and 2E, respectively).

As shown in Figure S2A (Additional file 3), none of the animals in which parental or p21-depleted SCP2 cells (eight per group) were injected into the mammary fat pad developed any bone lesions after two months, the date at which mice had to be sacrificed due to the tumor size. This timing may have been insufficient for tumor cells to grow into visible distant lesions in the mouse [37]. Thus, to investigate whether p21 is involved in the later stage of breast cancer progression, we examined its involvement in the development of bone osteolytic lesions using an intratibia injection model of parental and p21-deficient SCP2 cells in female Balb/c nude mice. By by-passing the early steps of metastasis, this experimental model allows for the assessment of tumor cell metastasis and survival in the bone marrow [38]. As shown in Figure S2B, C (Additional file 3), following X-ray examination of the bones, both groups of mice developed secondary tumors that caused severe osteolytic bone lesions, suggesting that p21 does not affect the later stages of bone metastasis. Collectively, these results indicate that while p21 is required for breast cancer cells to acquire an invasive phenotype, its effect is restricted to the earlier stages of tumor metastasis, namely induction of local cell invasion from the tumor to the surrounding tissues.

### TGFβ induces p21 expression in migratory and invasive human breast cancer cells

**Figure 3:**
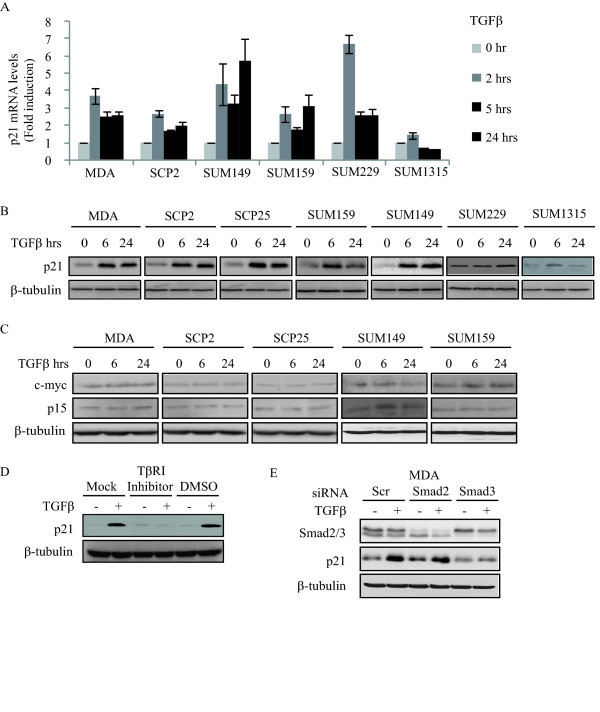
**TGFβ induces p21 expression in migratory and invasive human breast cancer cells**. **A**, Real-time PCR was performed to measure the mRNA level of p21 gene (error bars indicate SD; n = 3 independent experiments) for the indicated cell lines. **B**, Cells were treated with or without 5 ng/ml TGFβ for the indicated times. Total cell lysates were analyzed for p21 and β-tubulin protein levels by Western blotting. **C**, Total cell lysates were analyzed for c-myc, p15 and β-tubulin protein levels by Western blotting. **D**, SCP25 cells were pretreated with 10 µM TGFβ type I receptor (TβRI) inhibitor (SB431542) or vehicle (DMSO) for 30 minutes and then stimulated with TGFβ. Total cell lysates were analyzed for p21 and β-tubulin protein levels by Western blotting. **E**, MDA cells were transfected with 40 nM Scrambled (Scr), Smad2 or Smad3 siRNAs in response to TGFβ. Total cell lysates were analyzed for Smad2/3, p21 and β-tubulin protein levels by Western blotting.

p21 expression is tightly controlled by multiple signaling pathways [7]. Among these and of particular interest is the TGFβ/Smad signaling pathway [15,25,39]. Therefore, we examined the effect of TGFβ on the expression levels of p21 in several basal-like triple negative human breast cancer cell lines. These include the ductal adenocarcinoma MDA and its sub-progenies (SCP2 and SCP25) [40], an invasive ductal carcinoma SUM159PT (hereafter referred to as SUM159) derived from a patient with anaplastic carcinoma, an inflammatory invasive ductal carcinoma SUM149PT (SUM149), a pleural effusion derived SUM229PE (SUM229) and tumor cells derived from metastatic nodule of a patient with infiltrating ductal carcinoma SUM1315MO_2 _(SUM1315) [41]. As shown in Figure 3A, B, with the exception of SUM1315, TGFβ strongly induced p21 mRNA and protein levels in these cell lines. Interestingly, TGFβ showed no regulatory effect on the expression levels of other cell cycle regulatory genes, such as c-myc and p15 (Figure 3C), consistent with a loss of the TGFβ growth inhibitory responses in these cells (Figure 3C). Although p21 is a cell cycle inhibitor, the TGFβ-induced increases in p21 protein levels did not translate into growth inhibition by TGFβ (Additional file 4, Figure S3A), nor did it lead to G1 arrest in these breast cancer cells (Figure S3B). We next investigated the mechanisms by which TGFβ regulates p21 protein levels. As shown in Figure 3D, the TGFβ type I receptor (TβRI) inhibitor SB431542 blocked TGFβ-induced p21 protein expression, indicating that TGFβ regulation of p21 expression is mediated through the TGFβ receptor signaling cascade. Furthermore, we found this effect to be Smad-dependent and Smad3-specific, as TGFβ induced both phosphorylation of Smad2 and Smad3 (Additional file 4, Figure S3C), but was unable to induce p21 protein levels in MDA cells depleted of Smad3 but not of Smad2 (Figure 3E). Collectively, these data indicate that TGFβ potently induces p21 expression in a Smad3-dependent manner without affecting cell growth or cell cycle progression in invasive human basal-type breast cancer cells.

### p21 expression is required for TGFβ-mediated cell migration

**Figure 4:**
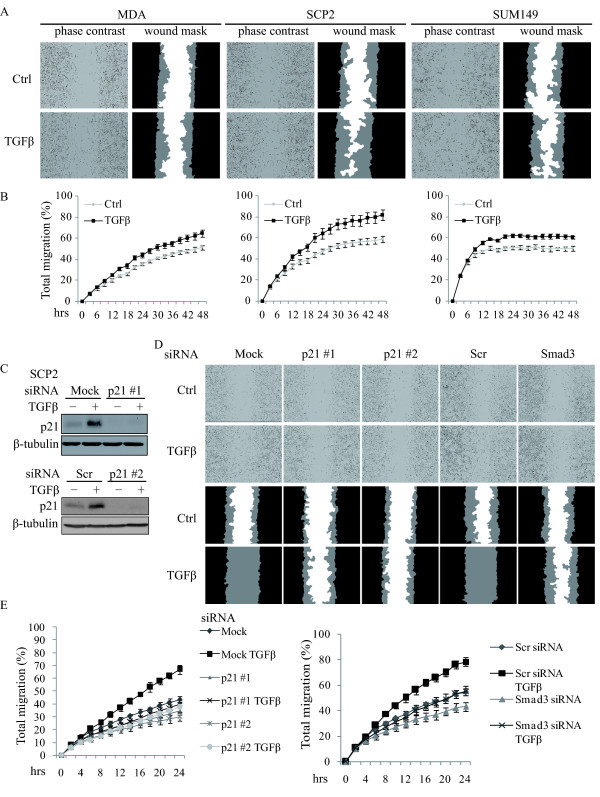
**p21 expression is required for TGFβ-mediated cell migration**. **A**, Representative images of phase contrast and wound mask of indicated cell lines stimulated with TGFβ in scratch/wound healing assay. The initial wound mask (black) and wound closure (grey) were measured using the Essen Instruments Scratch Wound Module. **B**, The time course of cell migration for the indicated cell lines was quantified using the relative wound density metrics at two-hour time intervals (error bars indicate SEM; n = 3 independent experiments). **C**, SCP2 cells were transfected with Scr or p21 siRNAs and then stimulated with or without TGFβ for 24 hrs. Total cell lysates were analyzed for p21 and β-tubulin by Western blotting. **D**, Representative images of phase contrast (top panels) and wound mask (bottom panels) of transfected SCP2 cells with the indicated siRNAs in scratch/wound healing assay. **E**, The time course of transfected SCP2 cell migration was quantified using the relative wound density metrics at two-hour time intervals (error bars indicate SEM; n = 3 independent experiments).

TGFβ is an important modulator of cell motility in breast cancer [25,42]. Thus, we investigated whether p21 could act downstream of TGFβ to promote cell migration. We first examined the effect of TGFβ on cell migration dynamics using the scratch/wound healing assay coupled to quantitative time-lapsed imaging (Essen IncuCyte™). Cell migration was measured by three integrated metrics: wound width, wound confluence and relative wound density, using the IncuCyte software. As shown in Figure 4A, B, TGFβ potently induced cell migration in MDA, SCP2 and SUM149. As a negative control, we also used SUM1315 in which TGFβ did not regulate p21 expression. As expected, there was no effect of TGFβ on cell migration in SUM1315 cells (Additional file 5, Figure S4A, B).

To then investigate whether p21 is required for TGFβ-induced cell migration, we knocked down p21 expression using two specific siRNAs in SCP2 cells and assessed the effect of TGFβ on cell migration dynamics by the scratch/wound healing assay. As shown in Figure 4C, TGFβ induced p21 expression in both mock and scrambled (Scr) siRNA transfected cells, while this effect was blocked in cells transfected with either p21 siRNAs, confirming the specificity and efficacy of our p21 siRNAs. Importantly, we found that while TGFβ potently induced cell migration in mock and Scr siRNA transfected SCP2 cells, this effect was completely blocked in cells in which p21 expression was depleted (Figure 4D, E). The effect of p21 siRNAs on TGFβ-induced cell migration was similar to that observed when cells were transfected with a siRNA against Smad3, used here as a positive control (Figure 4D, E). We also confirmed that these effects on cell migration were not secondary to changes in cell growth, as silencing of p21 expression had no effect on cell growth and proliferation (Figure S4C). These results demonstrate that TGFβ-mediated migration of human breast cancer cells is dependent on TGFβ-induced p21 expression.

### p21 expression is required for TGFβ-mediated cell invasion

**Figure 5:**
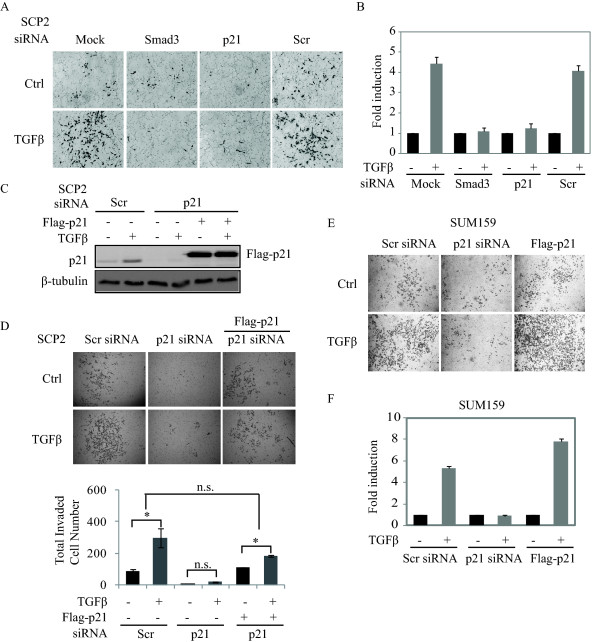
**p21 expression is required for TGFβ-mediated cell invasion**. **A**, SCP2 cells were transfected with the indicated siRNAs and cultured in the presence or absence of TGFβ. GFR-Matrigel coated Transwell invasion assay was performed and images of the invading cells were photographed. **B**, Total cell number was counted by Image J and fold induction was quantified (error bars indicate SD; n = 3 independent experiments). **C**, SCP2 cells were transfected with a Scr or p21 siRNA as well as a flag-tagged p21 cDNA in the presence or the absence of TGFβ. p21 protein levels were then analyzed by Western blotting. **D**, Cell invasion was assessed using the Transwell Invasion assay. The number of invaded cells was counted by Image J (error bars indicate SD; n = 3 independent experiments). **E**, Cell invasion of transfected SUM159 was assessed using the Transwell Invasion assay. **F**, Total cell number was counted by Image J and fold induction was quantified (error bars indicate SD; n = 3 independent experiments).

To examine the role of p21 in TGFβ-induced tumor cell invasion, SCP2 cells were transiently transfected with a Scr siRNA, a p21 siRNA or a Smad3 siRNA. The invasive potential of the cells was assessed using a GFR-Matrigel Transwell assay. As shown in Figure 5A, B, in mock and Scr siRNA transfected breast cancer cells, TGFβ significantly promoted cell invasion through the Matrigel and this effect was completely blocked in the absence of p21. Importantly, the inhibitory effect of the p21 siRNA on TGFβ-induced cell invasion was comparable to the effect of the Smad3 siRNA. To demonstrate the specificity of the p21 effect, we performed a rescue experiment. SCP2 cells in which endogenous p21 expression was silenced were transfected or not with a flag-tagged p21 cDNA (Figure 5C). In this setup, overexpression of the flag-p21 overrode the siRNA effect and restored p21 protein level (Figure 5C) as well as TGFβ-induced cell invasion through the GFR-Matrigel barrier (Figure 5D), indicating this effect is specifically mediated through p21. To avoid the limitation of the use of a single cell line, we also assessed the pro-invasive effect of p21 in SUM159 cells. Overexpressing or blocking p21 gene expression in these cells did not alter their growth in response to TGFβ (Additional file 6, Figure S5). Importantly, as shown in Figure 5E, F, we found SUM159 to be highly responsive to TGFβ-induced cell invasion. However, in the absence of p21 expression, the TGFβ pro-invasive effect was blocked, while overexpression of p21 potentiated this effect, similar to what was observed in SCP2 cells. Our results demonstrate that TGFβ-mediated migration and invasion of human breast cancer cells are dependent on TGFβ-induced p21 expression. Interestingly, the p21 effects are not limited to TGFβ signaling as blocking p21 expression also affected serum and EGF-induced cell invasion (Additional file 7, Figure S6). These results suggest that p21 plays a broad regulatory role in breast cancer cell invasion and may also explain the strong phenotype observed *in vivo*, on local tumor cell invasion, following p21 gene silencing (Figure 2C).

### p21 interacts with Smad3 and modulates TGFβ-induced transcriptional activity and downstream genes involved in cell invasion

**Figure 6:**
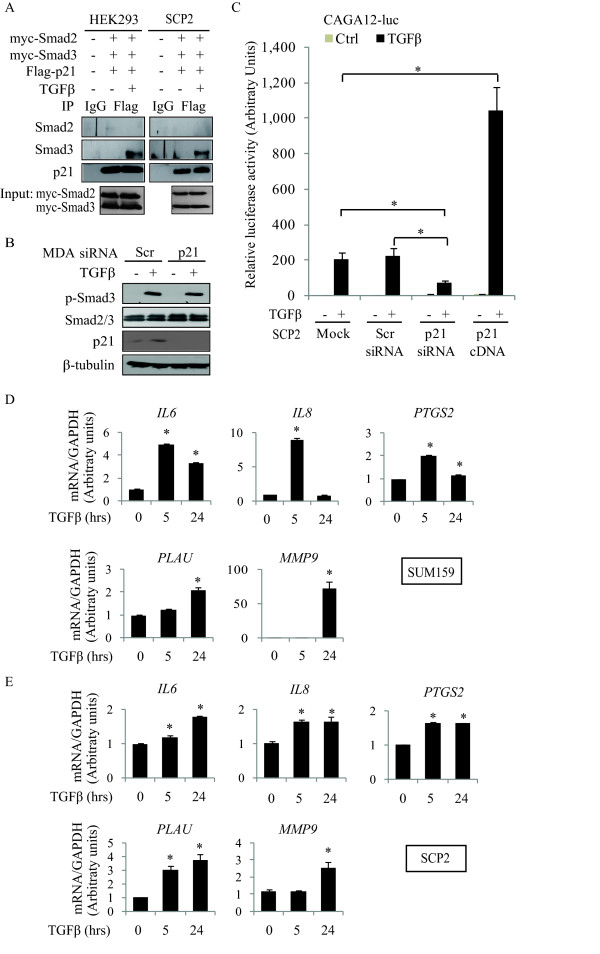
**p21 interacts with Smad3 and modulates TGFβ-induced transcriptional activity**. **A**, HEK293 and SCP2 cells were co-transfected with myc-Smad2, myc-Smad3 and flag-p21. Transfected cells were stimulated TGFβ for 8 hrs. Cell lysates were immunoprecipitated with an anti-flag antibody and analyzed by immunoblotting using Smad2/3 and p21 antibodies. **B**, Transfected MDA cells were immunoblotting by phospho-Smad3 (p-Smad3), Smad2/3 and β-tubulin antibodies in response to TGFβ for 30 minutes. **C**, SCP2 cells were co-transfected with either mock, Scr siRNA, p21 siRNA or flag-tagged p21 construct (p21 cDNA) and SBE promoter construct (CAGA12-luc). Transfected cells were stimulated with or without TGFβ for 16 hrs. Luciferase activity of CAGA12-luc was measured and normalized to β-galactosidase (error bars indicate SEM; n = 3 independent experiments). **D **and **E**, SUM159 and SCP2 cells were treated with or without TGFβ for the indicated times. The mRNA levels of indicated genes were then analyzed by real-time PCR (error bars indicate SEM; n = 3 independent experiments).

It has been previously shown that cytoplasmic p21 regulates actin cytoskeleton through binding and inhibiting ROCK1, resulting in decreased phosphorylation of actin-depolymerizing protein cofilin and increased cell migration in NIH3T3 fibroblasts and HeLa cells [43,44]. Therefore, we examined the phosphorylation and total protein expression levels of cofilin in breast cancer cells in response to TGFβ. As shown in Figure S7A (Additional file 8), TGFβ has no effect on the phosphorylation of cofilin. As cytoplasmic p21 contributes to regulate cofilin, we then examined the localization of p21 under the stimulation of TGFβ. Treatment with TGFβ caused accumulation of p21 in the nucleus in a time-dependent manner (Figure S7B). This suggests that TGFβ-induced and p21-driven cell migration and invasion in human breast cancer cells are not mediated through the ROCK/LIMK/cofilin pathway. Besides its function as a cell cycle regulator, p21 has also been shown to interact with multiple transcription factors [7] to selectively inhibit or induce expression of sets of genes involved in distinct biological functions, such as mitosis, DNA repair, survival and ECM components [45]. Thus, we investigated whether p21 could interact with the Smad proteins to regulate the TGFβ pro-invasive effects. Smad/p21 interactions were analyzed by co-immunoprecipitation studies in HEK293 and SCP2 cells co-transfected with myc-Smad2, myc-Smad3 and flag-p21. As shown in Figure 6A, while we could not detect any ligand-induced association between Smad2 and p21, we found TGFβ to clearly induce complex formation between Smad3 and p21 in the two cell lines. To assess the impact of the p21/Smad3 interaction on TGFβ signaling, we then examined the effect of p21 on TGFβ-induced Smad3 activity. As shown in Figure 6B, we found that knocking down p21 did not affect TGFβ-induced Smad3 phosphorylation. However, using a TGFβ/Smad transcriptional reporter construct (CAGA12-luc), we found that p21 is required for TGFβ-induced Smad transcriptional activity. Indeed, as shown in Figure 6C, p21 gene silencing abolished TGFβ-induced luciferase activity of the Smad reporter construct. Conversely, CAGA12-luc activity was markedly potentiated in SCP2 cells overexpressing p21 in response to TGFβ. These results indicate that TGFβ induces a complex formation between p21 and Smad3 and that while p21 does not affect the earlier stages of Smad3 activation (that is, phosphorylation), it is required for TGFβ-mediated Smad transcriptional activity.

We next performed gene profiling experiments in parental and p21-deficient SCP2 cells, using transiently transfected p21 siRNA as well as stably transfected p21 shRNA. Our arbitrary cutoff was set up at a minimum of two-fold induction. This led us to identify multiple p21-dependent TGFβ target genes, among which were selected those known to be associated with the tumor metastasis process. This shortlist included five candidate target genes: interleukin 6 (*IL6*), chemokine (*IL8*), prostaglandin-endoperoxide synthase 2 (*PTGS2*), plasminogen activator (*PLAU*) and matrix metalloproteinase (*MMP9*) [46]. To confirm that these genes were TGFβ downstream targets, SCP2 and SUM159 cells were stimulated or not with TGFβ and mRNA levels for these target genes were analyzed by quantitative real-time PCR (q-PCR). As shown in Figure 6D, E, TGFβ significantly increased the mRNA levels of *IL6*, *IL8*, *PTGS2*, *PLAU *and *MMP9 *in a time-dependent manner in both cell lines.

**Figure 7:**
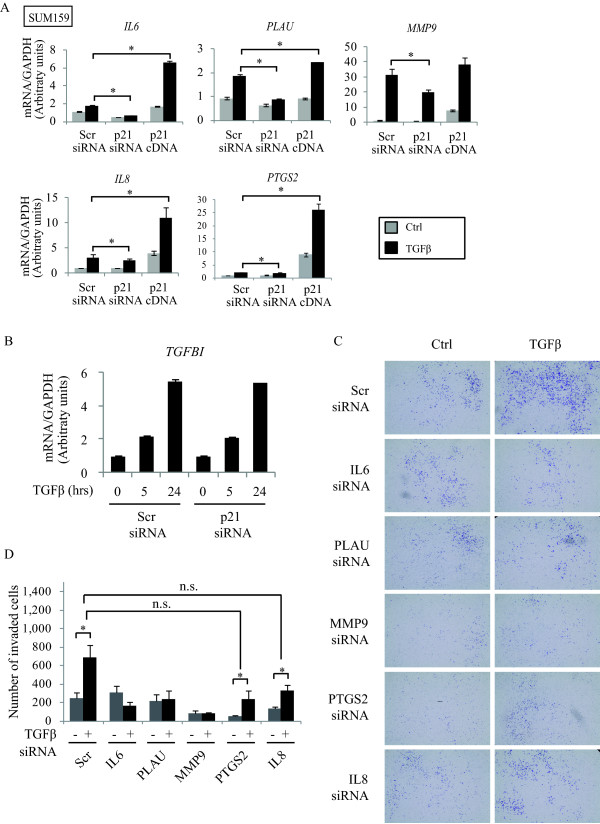
**p21 regulates TGFβ-induced downstream genes involved in cell invasion**. **A**, SUM159 cells were transfected with Scr or p21 siRNA as well as p21 cDNA. Cells were then treated with or without TGFβ and the mRNA levels of indicated genes were analyzed by real-time PCR (error bars indicate SEM; n = 3 independent experiments). **B**, SUM159 cells transfected with Scr siRNA and p21 siRNA were treated with TGFβ for the indicated times. The mRNA level of *TGFBI *gene was measured by real-time PCR (error bars indicate SEM; n = 3 independent experiments). **C**, SCP2 cells were transfected with the indicated siRNAs. Cell invasion was assessed using the Transwell Invasion assay. **D**, Total cell number was counted by Image J and number of invaded cells was quantified (error bars indicate SEM; n = 3 independent experiments).

To then address the role of p21 in the transcriptional regulation of these genes by TGFβ, we examined the effects of either silencing (using siRNA) or overexpressing p21 cDNA in SUM159 cells. As shown in Figure 7A, knocking down p21 gene expression blocked the TGFβ transcriptional regulation of *IL6*, *IL8*, *PLAU*, *MMP9 *and *PTGS2*, indicating that p21 is required for TGFβ to induce expression of these target genes. The same results were obtained in another breast cancer cell line (SCP2; data not shown). On the other hand, p21 overexpression in these cell lines potentiated the TGFβ transcriptional effects on these target genes. As a negative control and to ensure specificity of our results, we also analyzed the effect of silencing p21 on the TGFβ-mediated increase in transforming growth factor beta induced (*TGFBI*) mRNA. TGFβ regulated *TGFBI *mRNA independently of p21 (Figure 7B).

To address the contribution of these identified p21-dependent TGFβ target genes (*IL6*, *IL8*, *PLAU*, *MMP9 *and *PTGS2*) in regulating cell invasion, we silenced their gene expression using specific siRNAs. As shown in Figure 7C, D, inhibition of all five target genes impaired TGFβ-induced cell invasion, to a different extent. While depletion of *IL6*, *PLAU *and *MMP9 *drastically antagonized the TGFβ response, inhibition of *PTGS2 *and *IL8 *showed a moderate inhibitory effect. Moreover, examination of the siRNA effect on basal cell invasion indicated that *IL6 *and *PLAU *did not affect basal invasion, suggesting that they may be specifically required for the TGFβ pro-invasive response. On the other hand, inhibition of *MMP9*, *PTGS2 *and *IL8 *clearly affected basal cell invasion suggesting that these target genes have a broader effect on cell invasion, not limited to the TGFβ signaling pathway. Together, these results indicate that even though all five genes are important for TGFβ signaling leading to cell invasion, *IL6*, *PLAU *and *MMP9 *exert more predominant roles.

### p21/p/CAF regulates TGFβ transcriptional activity and Smad3 DNA binding

**Figure 8:**
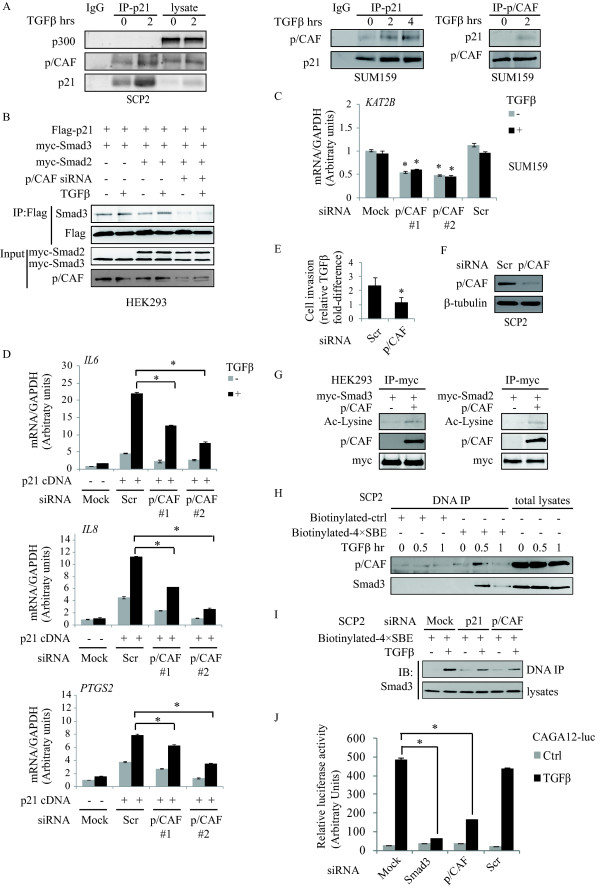
**p21/p/CAF regulates TGFβ transcriptional activity and Smad3 occupancy on SBE**. **A**, SCP2 and SUM159 cells were treated with TGFβ. Cell lysates were analyzed by co-immunoprecipitation using specific antibodies, as indicated. **B**, HEK293 cells were co-transfected with myc-Smad2, myc-Smad3 and flag-p21 with or without p/CAF siRNA. Transfected cells were stimulated with TGFβ for eight hours. Cell lysates were immunoprecipitated with an anti-flag antibody and analyzed by immunoblotting using Smad2/3 and flag antibodies. **C **and **D**, SUM159 cells were transfected with Scr or p/CAF siRNAs as well as a flag-tagged p21 cDNA, treated with or without TGFβ. The mRNA levels of indicated genes were analyzed by real-time PCR (error bars indicate SEM; n = 3 independent experiments). **E**, SCP2 cells transfected with Scr and p/CAF siRNA were stimulated with or without TGFβ. Cell invasion was quantified by relative TGFβ fold induction (error bars indicate SEM; n = 3 independent experiments). **F**, Transfected SCP2 cells were subjected to immunoblotting p/CAF and β-tubulin. **G**, HEK293 cells were co-transfected with myc-Smad3, myc-Smad2 and p/CAF. Immunoprecipitated Smad2/3 using an anti-myc antibody was subjected to Western blotting. **H**, DNA precipitation (DNA IP) was performed using biotinylated control and 4× CAGA SBE oligonucleotides, following by streptavidin precipitation. Western blotting of Smad3 and p/CAF is shown. **I**, SCP2 cells were transfected with p21 or p/CAF siRNAs. Samples were subjected to DNA IP and immunoblotting of Smad3. **J**, Transfected SCP2 cells were stimulated with or without TGFβ for 16 hrs. Luciferase activity of CAGA12-luc was measured and normalized to β-galactosidase (error bars indicated SEM; n =3 independent experiments).

p21 has been implicated in the control of gene transcription by associating with various transcription factors [7], but also regulates estrogen receptor-α-dependent gene expression by activating p300-CREBBP-driven (CBP) [47,48]. Gene transcription downstream of TGFβ signaling is also regulated by acetyltransferases, such as p300/CBP and p300/CBP-associated factor (p/CAF), a member of another HAT family, the so-called GCN5-related N-acetyl transferases [49-52]. Thus, we examined whether p21 could associate with either p300/CBP or p/CAF in response to TGFβ. Interestingly, we found that while TGFβ did not induce association between p21 and p300/CBP, it strongly induced complex formation between p21 and p/CAF in both SCP2 and SUM159 breast cancer cells (Figure 8A, left and right panels).

A previous report indicated that p/CAF directly binds to Smad3 [50]. As we have shown that TGFβ induces complex formation between Smad3 and p21 (Figure 6A), we investigated whether endogenous p/CAF is also required for Smad3 association with p21. For this, HEK293 cells were co-transfected with myc-Smad2, myc-Smad3 and flag-p21 with or without p/CAF siRNA to block expression of endogenous p/CAF. As shown in Figure 8B, TGFβ induced complex formation between p21 and Smad3, independently of Smad2. Interestingly, depletion of p/CAF completely prevented this interaction, indicating that endogenous p/CAF is required for Smad3 interaction with p21.

To investigate whether p/CAF is necessary for the regulation of p21-dependent TGFβ downstream target genes, SUM159 cells were transiently transfected with flag-tagged p21 in the presence or the absence of two different p/CAF siRNAs. The gene expression of p/CAF (also known as K(lysine) acetyltransferase 2B, KAT2B) was measured to verify the efficiency of p/CAF knockdown by q-PCR (Figure 8C). Overexpression of p21 potentiated induction of *IL6*, *IL8 *and *PTGS2 *mRNA by TGFβ. However, these effects were significantly blocked when p/CAF gene expression was silenced, indicating that p/CAF is required for p21-dependent gene expression of the TGFβ targets (Figure 8D). The requirement of p/CAF downstream of TGFβ was further investigated using the Transwell Matrigel assay. As shown in Figure 8E, knocking down p/CAF gene expression significantly impaired TGFβ-induced cell invasion. Efficiency of the siRNA was verified by Western blotting (Figure 8F).

Because acetyltransferase p/CAF regulates gene transcription by acetylating histones and transcription factors [49], we then assessed whether TGFβ could induce global changes in histone acetylation in breast cancer cells. For this, total histone proteins were extracted from SCP2 cells, treated or not with TGFβ and subjected to immunoblotting using an acetylated lysine antibody. As shown in Figure S8 (Additional file 9), TGFβ had no effect on global histone acetylation while TSA, a histone deacetylase inhibitor, showed a marked increase in the acetylation levels. This suggested that the functional relevance of the p/CAF recruitment to the p21/Smad complex may be more directed towards acetylation of specific targets rather than global histone modifications. To address this, we examined whether p/CAF could acetylate p21 and/or the Smads. Interestingly, we found that p/CAF is capable of interacting with Smad2 and Smad3, leading to an increased acetylation of both Smad proteins (Figure 8G). Moreover, the acetylation is specific to Smad2 and Smad3, as p21 did not show any increased acetylation by p/CAF (data not shown). Smad3 acetylation has been suggested to be required for its DNA binding activity [51]. Thus, this led us to investigate whether p/CAF could associate with DNA-bound Smad3, by DNA immunoprecipitation (DNA IP) using biotinylated control and biotinylated Smad binding element (4× CAGA) DNA probes. As shown in the Figure 8H, we found TGFβ to specifically induce binding of both Smad3 and p/CAF to DNA. Furthermore, we found that gene silencing of p/CAF and p21, using siRNAs, prevented Smad3 binding to the SBE (Figure 8I), suggesting that both p21 and p/CAF are required for Smad3 DNA binding and Smad3-mediated transcriptional activity. Having shown that p21 is indeed required for Smad3-mediated transcriptional activity (Figure 6C), we then assessed the effect of knocking down p/CAF on Smad3 transcriptional activity using the CAGA12-luc reporter construct. As shown in Figure 8J, the results clearly indicate that p/CAF is required for TGFβ-induced Smad3 transcriptional activity.

Collectively, these data indicate that p21 and p/CAF regulate TGFβ transcriptional activity by controlling Smad3 occupancy on its DNA binding elements. TGFβ induces a complex formation between Smad3, p21 and p/CAF, further leading to Smad3 acetylation by p/CAF. Furthermore, both p21 and p/CAF are required for Smad3 DNA binding and Smad3-mediated transcriptional activity, highlighting a novel mechanism by which the p21/p/CAF/Smad3 complex contribute to the activation of TGFβ target gene transcription.

### High expression of p/CAF/p21/p-Smad3 is associated with lymph node positivity

**Figure 9:**
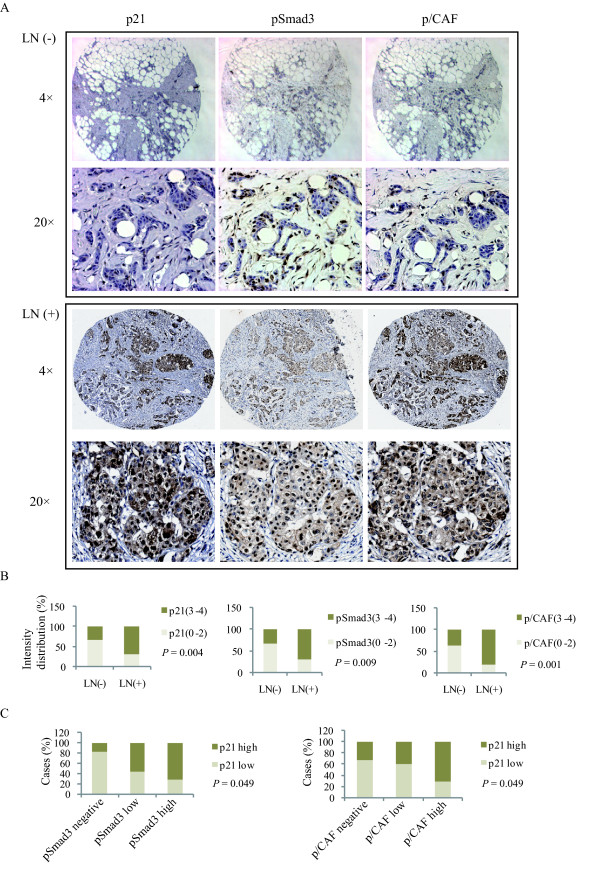
**High expression of p/CAF/p21/pSmad3 is associated with lymph node positivity**. **A**, Representative immunohistochemistry images of pSmad3, p21 and p/CAF in breast cancer tissue microarray samples. **B**, Overall pSmad3, p21 and p/CAF immunohistochemistry staining intensity between lymph node negative (LN-) and positive (LN+) tissues. **C**, Percentage of case distribution according to immunoreactivity of pSmad3, p21 and p/CAF in tumor cells, the score for negative (0), low (1 to 2) and high (3 to 4).

Lymph node involvement is an important prognostic indicator in clinical breast cancer outcomes [53]. High expression of TGFβ1 is correlated with a high incidence of lymph node metastasis [54]. To examine the association among active TGFβ/Smad signaling, p21 and p/CAF with lymph node metastasis, we performed immunohistochemistry to measure the expression levels of serine 423/425 phosphorylated Smad3 (pSmad3), p21 and p/CAF in tissue microarray containing 50 invasive ductal breast tumors, 25 of which are lymph node positive. The immunoreactivity for pSmad3, p21 and p/CAF protein expression in tumor cells was graded and described in Methods. We considered a score of 0 to 2 as a low phosphorylation/expression level and a score of 3 to 4 as a high phosphorylation/expression level. As shown in Figure 9A, B, lymph node negative patients showed low levels of pSmad3, p21 and p/CAF expressions, whereas a significant enrichment of high pSmad3 (68%), p21 (68%) and p/CAF (80%) was observed in patients with positive lymph nodes (*P *= 0.009, *P *= 0.004, and *P *= 0.001, respectively). To distinguish between tumor cells and stroma, we used a cytokeratin antibody (AE1/AE3), a cell marker specific to neoplastic cells of epithelial origin. As shown in Figure S9 (Additional file 10), p21 is specifically expressed at a higher level in breast tumor cells but not in stroma cells. We also examined the relationships between pSmad3 levels and p/CAF protein expression with p21 protein levels, using the Pearson's correlation test. As shown in Figure 9C, our results clearly indicate that high levels of phosphorylated Smad3 and p/CAF expression significantly correlate with high p21 protein expression. Collectively, these data indicate that active TGFβ/Smad3 signaling is associated with high p21 and p/CAF protein expression levels and significantly correlates with lymph node metastasis in breast cancer.

## Discussion

While p21 was initially characterized as an important cell cycle inhibitor, recent studies suggest that cytoplasmic p21 has anti-apoptotic and actin cytoskeleton regulatory functions [27,43,55]. The accumulation of cytoplasmic p21 is associated with Ras and HER2/neu activated tumorigenic transformation [32,44]. Moreover, overexpression of p21 is associated with poor prognosis of many types of cancer. However, the function of p21 in breast cancer has not been established. In our study, we assessed p21 levels with clinical outcomes in breast cancer patients. High p21 expression correlates with poor overall survival and distant metastasis free survival. Furthermore, using an *in vivo *model of mammary fat pad transplantation of metastatic human breast cancer cells in mice, we showed that while silencing p21 gene expression did not affect the primary tumor formation, it potently prevented primary tumor cells to invade into surrounding tissues. Together, our results provide evidence of a tumor-promoting role for p21 in primary tumor local invasion.

Previous studies have indicated that during breast cancer progression, TGFβ cytostatic responses are lost while pro-migratory and pro-invasive effects are maintained [56]. Here, we found that all invasive breast cancer cell lines tested were resistant to growth inhibition by TGFβ and that while TGFβ did not induce any change in p15 or c-myc expression levels, it strongly up-regulated p21 expression arguing that in advanced breast cancer p21 functions independently of cell cycle regulation. This is in contrast to the effect observed in human immortalized the keratinocyte cell line HaCaT, where TGFβ-mediated p21 gene expression leads to cell cycle arrest [39]. Indeed, we found that the induction of p21 in invasive breast cancer cells is required for the pro-migratory and pro-invasive effects of TGFβ. In accordance with these results, depletion of p21 did not modulate primary tumor growth *in vivo *but strongly blocked tumor invasion capacity. These findings together support the notion of a direct oncogenic role for p21 in breast cancer progression.

We further report that the TGFβ-mediated increase in p21 expression is Smad-dependent and Smad3 specific. This is interesting in light of previous reports indicating that overexpression of a dominant negative form of Smad3 reduced the ability of cancer cells to metastasize [57] and that Smad3, but not Smad2, promotes breast cancer metastasis in mice [58]. Furthermore, while Smad2 mutations in cancer have been described, no mutations in Smad3 or p21 have yet been reported. Together these data suggest that in breast cancer Smad3 pro-invasive functions are mediated by p21 and that targeting p21 may prove useful to improve the clinical course of metastatic patients.

**Figure 10:**
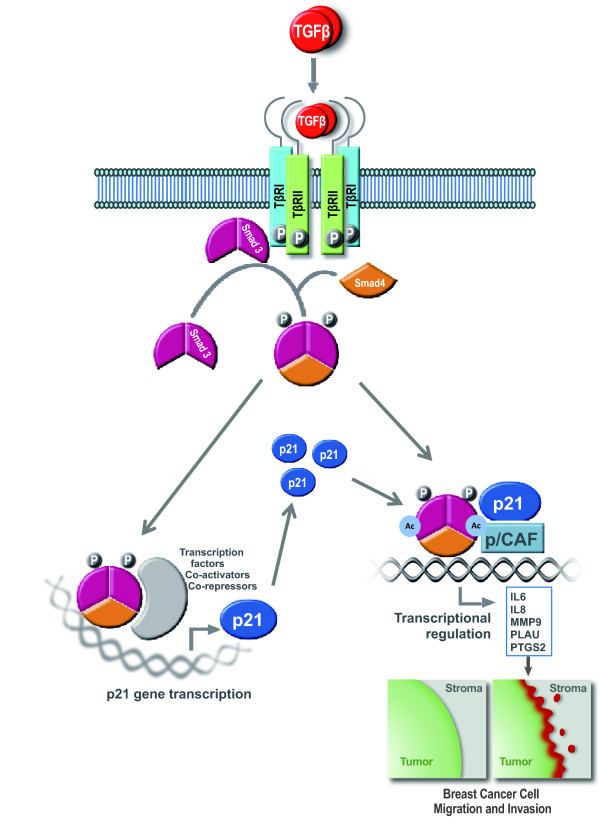
**Model of pro-invasive function for the cell cycle regulator p21 in human breast cancer**. The role of p21 as both a direct transcriptional target of TGFβ and a co-stimulatory factor of p/CAF/Smad3 in regulation of pro-invasive genes in triple negative breast cancer cells.

Tumor cell migration and invasion are critical initiation steps in the process of breast cancer metastasis. It has been suggested that cytoplasmic p21 regulates ROCK/LIMK/cofilin pathway to promote cell migration; however, we found that TGFβ had no effect on regulating cofilin activity in breast cancer cells. In our studies, we identified a novel role for p21 in the transcriptional regulation of TGFβ/Smad3 signaling through the interaction of p21 and Smad3 in invasive breast cancer cells. The interaction between p21 and Smad3 was p/CAF-dependent, but whether this interaction is direct will require further investigation. Furthermore, the effects of p21 on cell migration and invasion are mediated through interactions with Smad3 and p/CAF, which in turn modulate Smad3 acetylation, DNA binding and transcriptional activity, as well as gene transcription of several TGFβ pro-invasive downstream target genes. It will be interesting to further investigate whether p21 is selective for the pro-oncogenic activity of TGFβ or whether it is also required for the transcriptional regulation of other types of TGFβ responses and target genes. Taken together, our results demonstrate that p21 is both a direct transcriptional target of TGFβ and a co-stimulatory factor of Smad3 in regulation of pro-invasive genes in breast cancer cells (Figure 10).

Finally, we investigated the clinical relevance of TGFβ-mediated p21/p/CAF pathway in breast cancer. The prognosis of breast carcinomas is related to various clinical and pathological parameters. Axillary lymph node metastasis is one of the most important prognostic parameters in the absence of distant metastasis. There is a sharp difference in survival rate between patients with positive and negative lymph nodes. In our studies, we found a significant association of active TGFβ/Smad3 signaling, p21 and p/CAF expression with lymph node positivity, making them potential useful prognosis markers for lymph node metastasis.

## Conclusion

In this study, we described a pro-invasive function for the cell cycle regulator p21 in human breast cancer. High expression of p21 positively correlated with poor overall and distant metastasis-free survival outcomes in breast cancer patients. We identified p21 as a novel downstream regulator of TGFβ-mediated breast cancer cell migration and invasion. We found p21 to interact with Smad3 and the acetyltransferase p/CAF and to regulate the Smad transcriptional activity, as well as gene transcription of several TGFβ-induced pro-metastatic genes. These results highlight an important role for p21/p/CAF in TGFβ-induced breast cancer cell migration and invasion at the transcriptional level.

## Abbreviations

BSA: bovine serum albumin; DMEM: Dulbecco's modified Eagle's medium; DMFS: distant metastasis free survival; DMSO: dimethyl sulfoxide; ECM: extracellular matrix; p15: p15Ink4b; EGF: epidermal growth factor; ER: estrogen receptor; FBS: fetal bovine serum; GFR: growth factor reduced; IL6: interleukin 6; MMP9: matrix metalloproteinase 9; MTT: thiazolyl blue tetrazolium bromide; OS: overall survival; p21: p21Cip1; p/CAF: p300/CBP-associated factor; PLAU: plasminogen activator; PTGS2: prostaglandin-endoperoxide synthase 2; SDS: sodium dodecyl sulphate; TβRI: TGFβ type I receptor; TGFβ: transforming growth factor-beta; TSA: trichostatin A

## Competing interests

The authors declare that they have no competing interests.

## Authors' contributions

MD and JJL designed the experiments and wrote the manuscript. SAR and SA assisted in editing the manuscript. MD and JJL were involved in all the experiments, data analysis and interpretation. AAA analyzed the tissue microarray data and tumor local invasiveness. SAR and AA performed *in vivo *studies and analyzed the mammary tumor growth. All authors read and approved the final manuscript.

## Supplementary Material

Additional file 1 **Table S1: Patient characteristics from breast carcinoma microarray tissue sections**. The patient characteristics including tumor grade, TNM and lymph node involvement are provided for breast carcinoma microarray slides (BCR961 and T088, Biomax). Click here for file

Additional file 2 **Figure S1: The relationship between p21 expression and clinical outcomes in ER+ and ER- breast cancer patients**. **A **and **B**, The correlation of p21 expression with overall survival and distant metastasis free survival in ER+ and ER- breast cancer patients was assessed by Kaplan-Meier survival analysis. The patients were divided into high or low groups based on the median of p21 expression. Number of breast cancer patients at risk with higher expression (red) and lower expression (black) of p21 at the indicated time points. Click here for file

Additional file 3 **Figure S2: Knocking down p21 does not affect bone osteolytic lesions**. Representative radiographs of skeletal lesions in two groups of mice (parental and shRNA p21) were taken by X-ray using Faxitron. **A**, parental and shRNA p21 SCP2 cells were injected in orthotopic site (mammary gland). **B **and **C**, parental and shRNA p21 SCP2 cells were injected in tibia. The lesions are highlighted by arrows. The X-ray scores of bone lesions at Week 8 are shown for the two groups of animals. Data are reported as the mean ± SEM of eight animals in each group and the X-ray scoring differences were tested by an independent two-sample t-test. No statistically significant difference was observed between the two groups (*P *= 0.81). *P *< 0.05 was considered to be statistically significant. Click here for file

Additional file 4 **Figure S3: TGFβ has no effect on cell growth or cell cycle progression in basal-type triple negative breast cancer cells**. **A**, The indicated cell lines were stimulated with TGFβ for 48 hrs and subjected to MTT assay (error bars indicate SD; n = 3 independent experiments). **B**, SCP2 cells were incubated with or without TGFβ for the indicated times and cell cycle distribution was determined by flow cytometry. **C**, MDA cell lysates were analyzed for phospho-Smad2, phospho-Smad3 and total Smad2/3 protein levels by Western blotting. Click here for file

Additional file 5 **Figure S4: Effect of TGFβ on cell migration in SUM1315**. **A**, Representative images of phase contrast and wound mask of SUM1315 cell line stimulated with TGFβ in scratch/wound healing assay. **B**, The time course of cell migration for SUM1315 was quantified using the relative wound density metrics (error bars indicate SEM; n = 3 independent experiments). **C**, Cell viability (MTT) assay of transfected SCP2 cells subjected to scratch/wound healing assay. Click here for file

Additional file 6 **Figure S5: Effect of p21 expression on cell proliferation**. SUM159 cells were transfected with a Scr siRNA, a p21 siRNA and a flag-tagged p21 construct. Transfected SUM159 cells were then subjected to MTT assay (error bars indicate SD, n = 3). Click here for file

Additional file 7 **Figure S6: Effect of p21 depletion on serum and EGF-stimulated cell migration**. SCP2 cells were transfected with or without p21 siRNA. Cell invasion was assessed using the Transwell Invasion assay. Total number of cells invaded was counted by Image J (error bars indicate SEM; n = 3 independent experiments). * *P *< 0.05, n.s., non significant. Click here for file

Additional file 8 **Figure S7: Effect of TGFβ on cofilin phosphorylation and p21 nuclear localization**. **A**, SCP2 and SUM159 cells were treated with or without 5 ng/ml TGFβ for the indicated times. Total cell lysates were analyzed for phospho-cofilin and colifin protein levels by Western blotting. **B**, Immunocytochemistry was performed using p21 (green) antibody and DAPI (blue). The scale bar is 10 µm. Click here for file

Additional file 9 **Figure S8: Effect of TGFβ on acetylation of total histone proteins**. SCP2 cells were treated with TGFβ and TSA for the indicated times. Immunoblots of total histone proteins using an acetylated lysine (Ac-Lysine) antibody. Click here for file

Additional file 10 **Figure S9: p21 is specifically overexpressed in breast tumor cells**. Representative immunohistochemistry images of AE1/AE3 and p21 in lymph node negative (LN-) and positive (LN+) of breast cancer tissue microarray samples. Click here for file
