# Erratum to: A novel function for p21 Cip1 and acetyltransferase p/CAF as critical transcriptional regulators of TGFβ-mediated breast cancer cell migration and invasion

**DOI:** 10.1186/s13058-017-0832-7

**Published:** 2017-03-28

**Authors:** Meiou Dai, Amal A. Al-Odaini, Ani Arakelian, Shafaat A. Rabbani, Suhad Ali, Jean-Jacques Lebrun

**Affiliations:** Division of Medical Oncology, Department of Medicine, McGill University Health Center, Royal Victoria Hospital, Montreal, Canada

## Erratum

After publication of this work [1] an error was noticed in Fig. 3b. The tubulin Western blot control was accidentally used for both SCP2 and SCP25 cells. The corrected figure is shown below. We apologize for this error, which did not affect any of the interpretations or conclusions of the article.

**Fig. 3:**
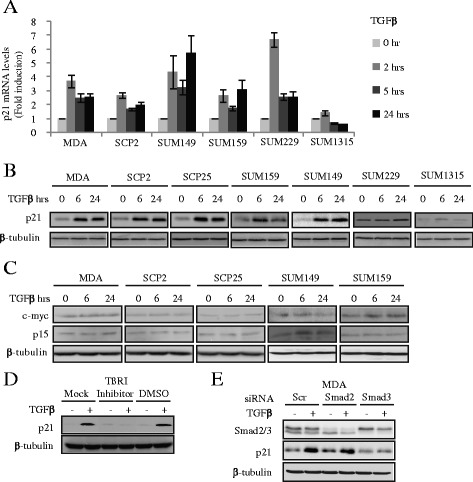
TGFβ induces p21 expression in migratory and invasive human breast cancer cells. **a**, Real-time PCR was performed to measure the mRNA level of p21 gene (error bars indicate SD; *n* = 3 independent experiments) for the indicated cell lines. **b**, Cells were treated with or without 5 ng/ml TGFβ for the indicated times. Total cell lysates were analysed for p21 and β-tubulin protein levels by western blotting. **c**, Total cell lysates were analysed for c-myc, p15 and β -tubulin protein levels by western blotting. **d**, SCP25 cells were pre-treated with 10 μM TGFβ type I receptor (TßRI) inhibitor (SB431542) or vehicle (DMSO) for 30 min and then stimulated with TGFβ. Total cell lysates were analysed for p21 and β-tubulin protein levels by western blotting. **e**, MDA cells were transfected with 40 nM Scrambled (Scr), Smad2 or Smad3 siRNAs in response to TGFβ. Total cell lysates were analysed for Smad2/3, p21 and β-tubulin protein levels by western blotting

## References

[1] Dai M (2012). A novel function for p21Cip1 and acetyltransferase p/CAF as critical transcriptional regulators of TGFbeta-mediated breast cancer cell migration and invasion. Breast Cancer Res.

