# Supplementary figures and images for: A novel function for p21Cip1 and acetyltransferase p/CAF as critical transcriptional regulators of TGFβ-mediated breast cancer cell migration and invasion
Source: Breast Cancer Res. 2012 Sep 20;14(5):R127. doi: 10.1186/bcr3322 (PMC4053104; doi:10.1186/bcr3322)

A

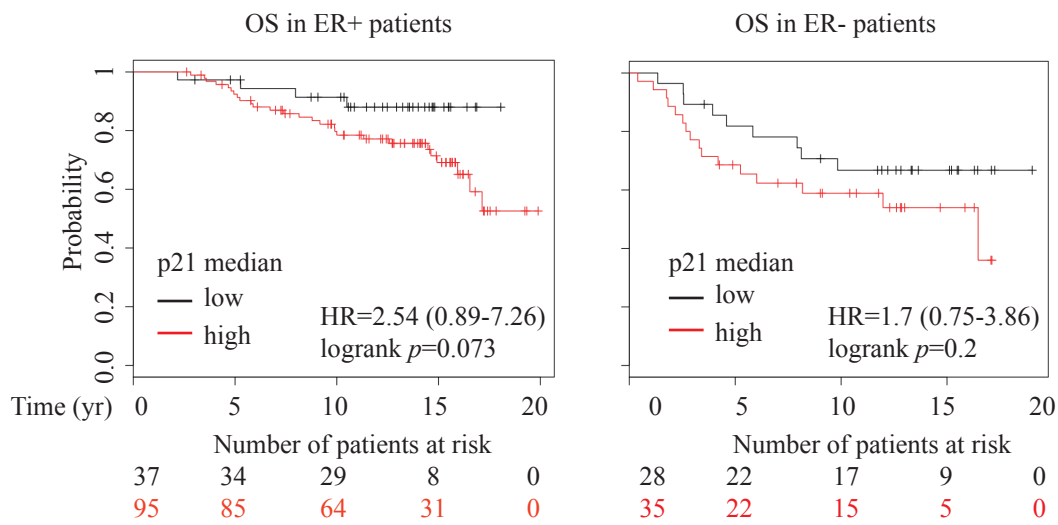

B

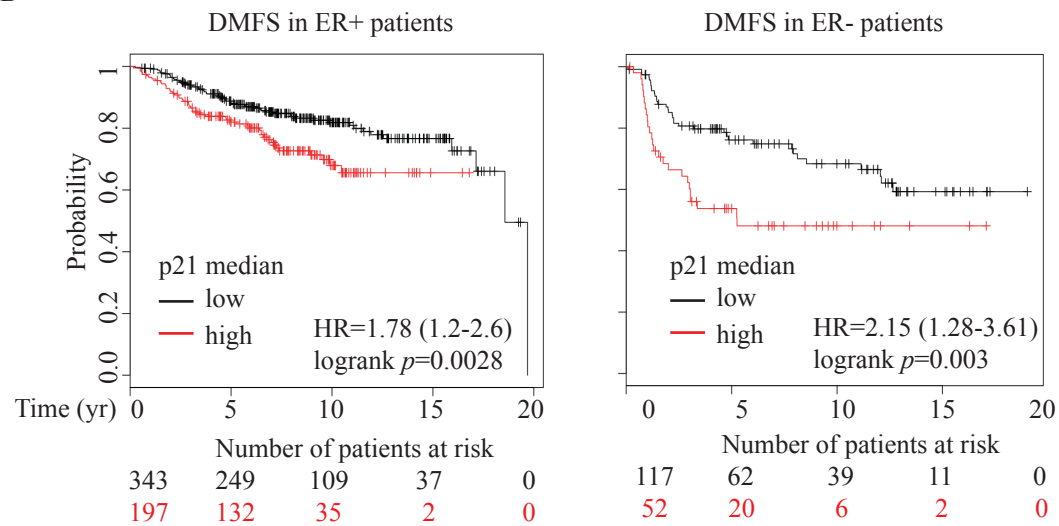

Figure S1

Supplement: Additional file 2 — Figure S1: The relationship between p21 expression and clinical outcomes in ER+ and ER- breast cancer patients. A and B, The correlation of p21 expression with overall survival and distant metastasis free survival in ER+ and ER- breast cancer patients was assessed by Kaplan-Meier survival analysis. The patients were divided into high or low groups based on the median of p21 expression. Number of breast cancer patients at risk with higher expression (red) and lower expression (black) of p21 at the indicated time points. [file bcr3322-S2.PDF]

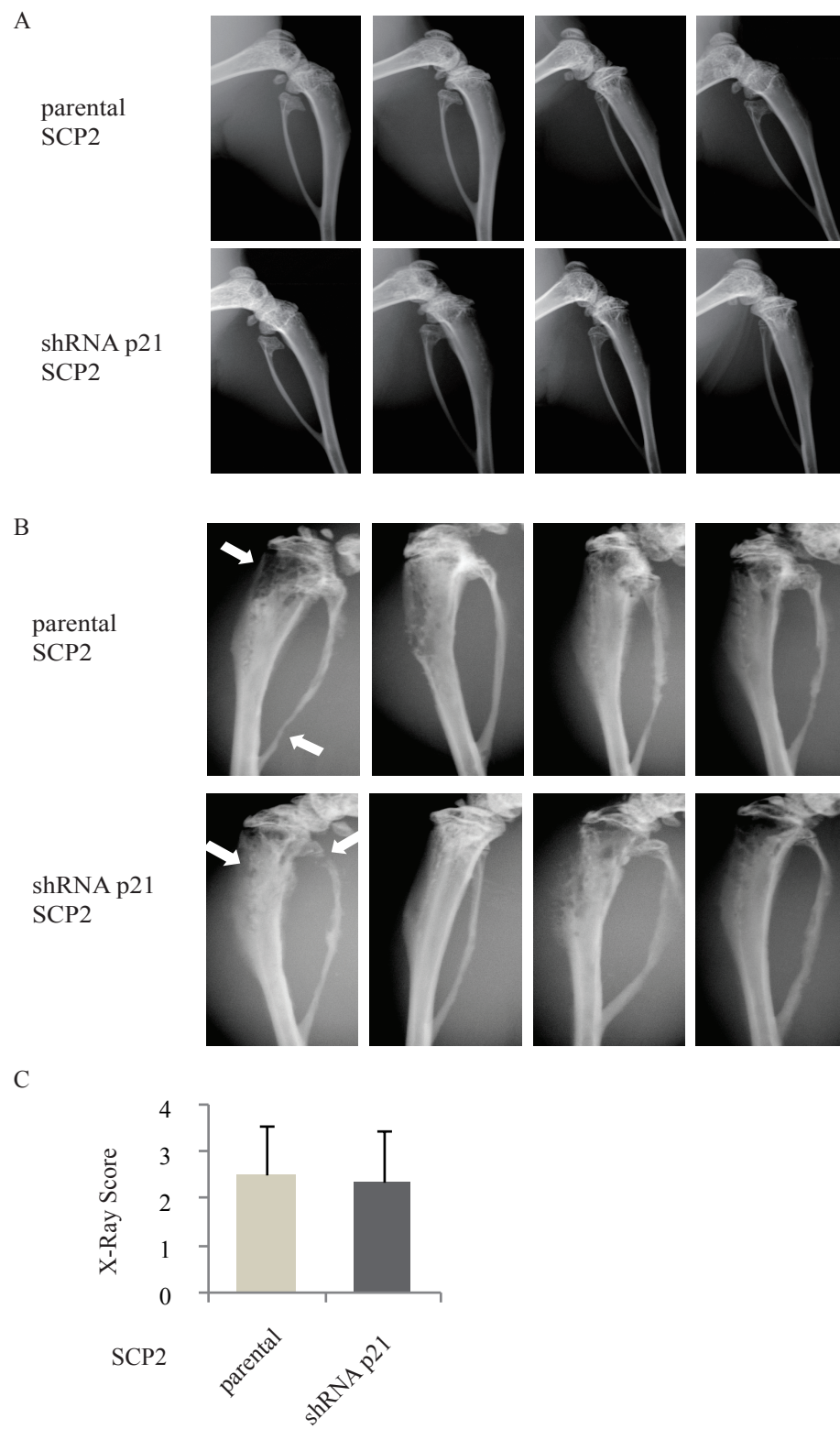

Figure S2

Supplement: Additional file 3 — Figure S2: Knocking down p21 does not affect bone osteolytic lesions. Representative radiographs of skeletal lesions in two groups of mice (parental and shRNA p21) were taken by X-ray using Faxitron. A, parental and shRNA p21 SCP2 cells were injected in orthotopic site (mammary gland). B and C, parental and shRNA p21 SCP2 cells were injected in tibia. The lesions are highlighted by arrows. The X-ray scores of bone lesions at Week 8 are shown for the two groups of animals. Data are reported as the mean ± SEM of eight animals in each group and the X-ray scoring differences were tested by an independent two-sample t-test. No statistically significant difference was observed between the two groups (P = 0.81). P < 0.05 was considered to be statistically significant. [file bcr3322-S3.PDF]

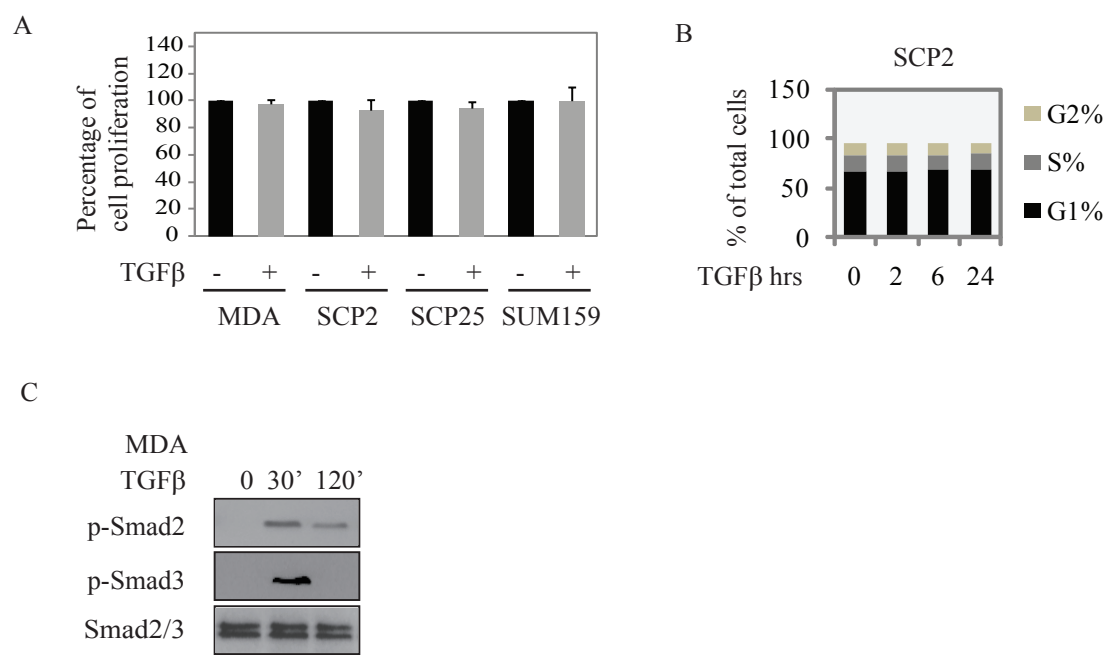

Figure S3

Supplement: Additional file 4 — Figure S3: TGFβ has no effect on cell growth or cell cycle progression in basal-type triple negative breast cancer cells. A, The indicated cell lines were stimulated with TGFβ for 48 hrs and subjected to MTT assay (error bars indicate SD; n = 3 independent experiments). B, SCP2 cells were incubated with or without TGFβ for the indicated times and cell cycle distribution was determined by flow cytometry. C, MDA cell lysates were analyzed for phospho-Smad2, phospho-Smad3 and total Smad2/3 protein levels by Western blotting. [file bcr3322-S4.PDF]

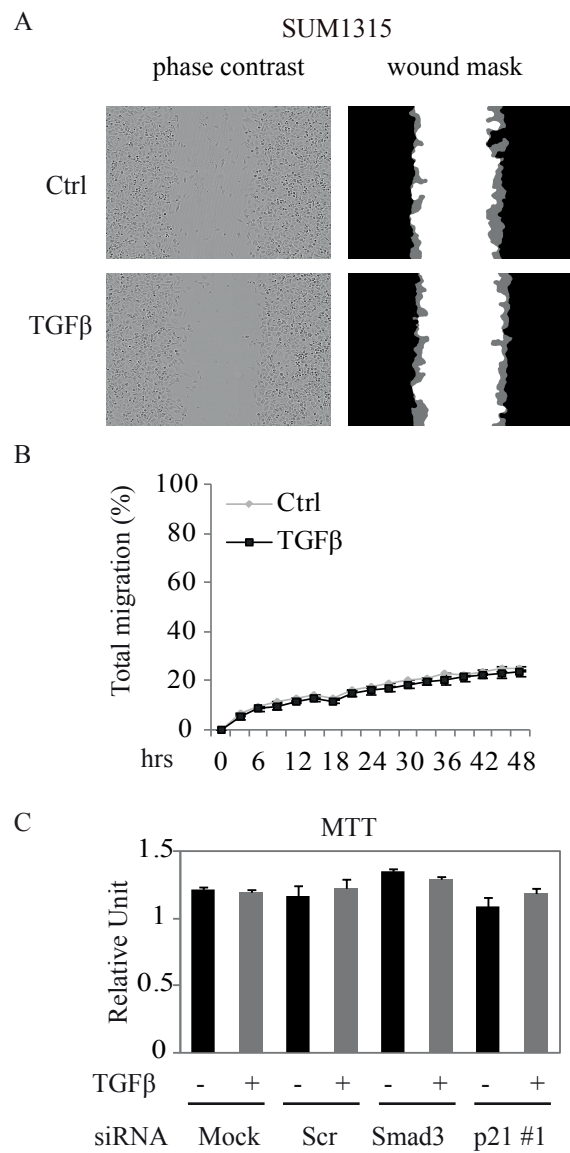

Figure S4

Supplement: Additional file 5 — Figure S4: Effect of TGFβ on cell migration in SUM1315. A, Representative images of phase contrast and wound mask of SUM1315 cell line stimulated with TGFβ in scratch/wound healing assay. B, The time course of cell migration for SUM1315 was quantified using the relative wound density metrics (error bars indicate SEM; n = 3 independent experiments). C, Cell viability (MTT) assay of transfected SCP2 cells subjected to scratch/wound healing assay. [file bcr3322-S5.PDF]

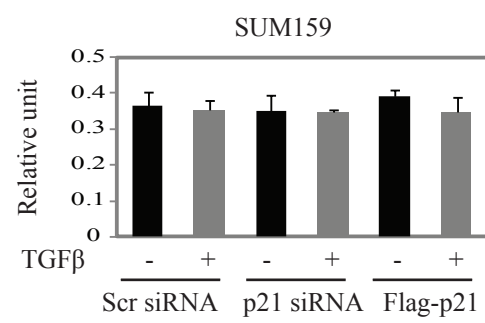

Figure S5

Supplement: Additional file 6 — Figure S5: Effect of p21 expression on cell proliferation. SUM159 cells were transfected with a Scr siRNA, a p21 siRNA and a flag-tagged p21 construct. Transfected SUM159 cells were then subjected to MTT assay (error bars indicate SD, n = 3). [file bcr3322-S6.PDF]

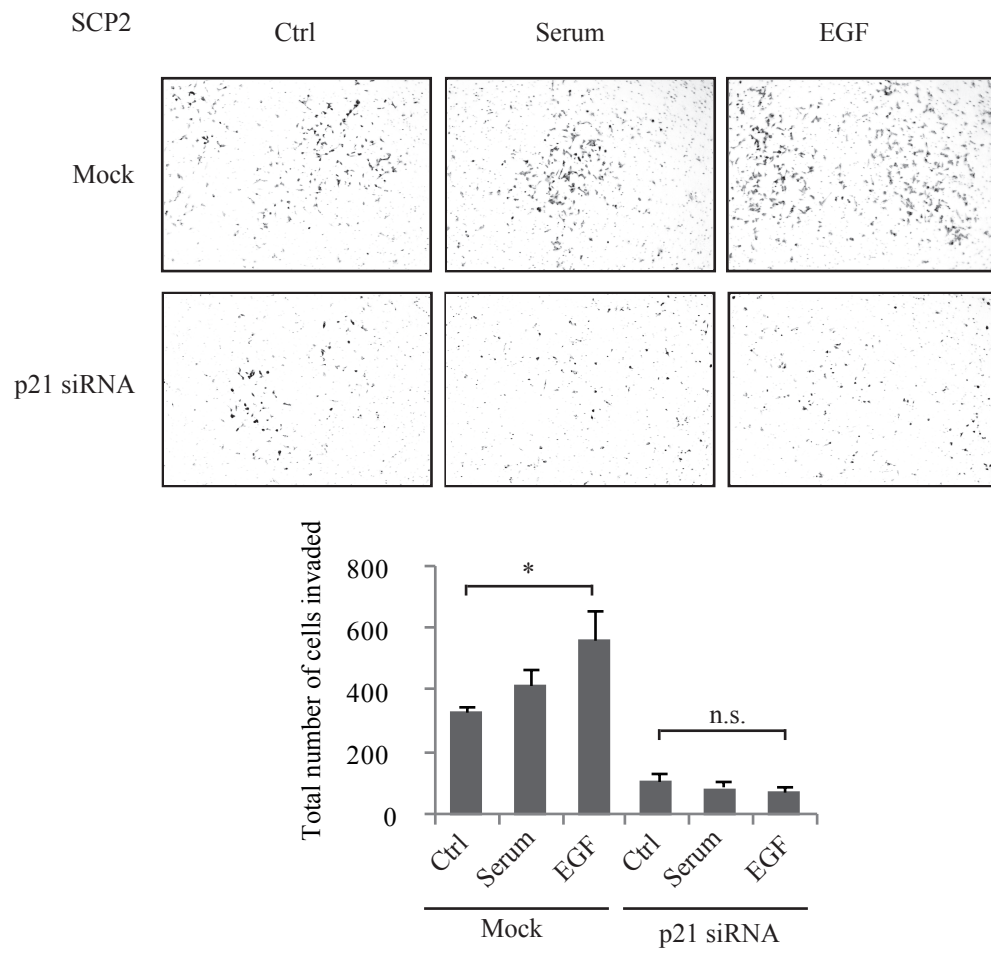

Figure S6

Supplement: Additional file 7 — Figure S6: Effect of p21 depletion on serum and EGF-stimulated cell migration. SCP2 cells were transfected with or without p21 siRNA. Cell invasion was assessed using the Transwell Invasion assay. Total number of cells invaded was counted by Image J (error bars indicate SEM; n = 3 independent experiments). * P < 0.05, n.s., non significant. [file bcr3322-S7.PDF]

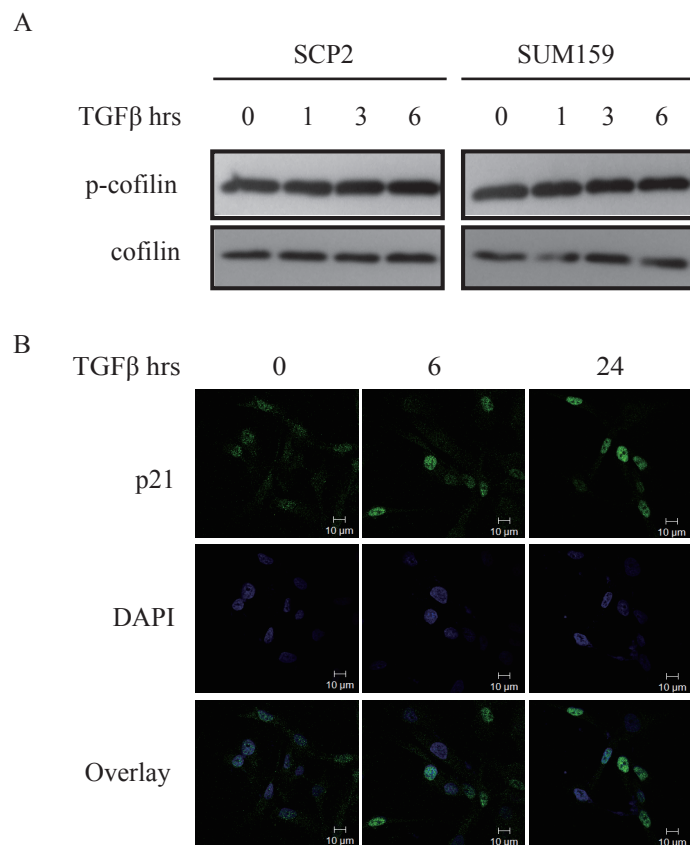

Figure S7

Supplement: Additional file 8 — Figure S7: Effect of TGFβ on cofilin phosphorylation and p21 nuclear localization. A, SCP2 and SUM159 cells were treated with or without 5 ng/ml TGFβ for the indicated times. Total cell lysates were analyzed for phospho-cofilin and colifin protein levels by Western blotting. B, Immunocytochemistry was performed using p21 (green) antibody and DAPI (blue). The scale bar is 10 µm. [file bcr3322-S8.PDF]

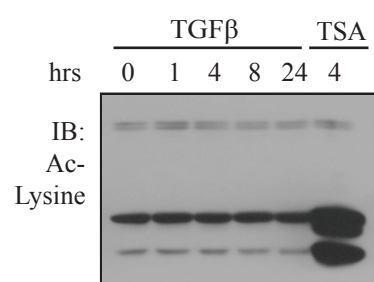

Figure S8

Supplement: Additional file 9 — Figure S8: Effect of TGFβ on acetylation of total histone proteins. SCP2 cells were treated with TGFβ and TSA for the indicated times. Immunoblots of total histone proteins using an acetylated lysine (Ac-Lysine) antibody. [file bcr3322-S9.PDF]

AE1/AE3

p21

LN (-)

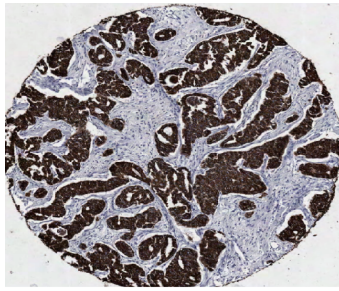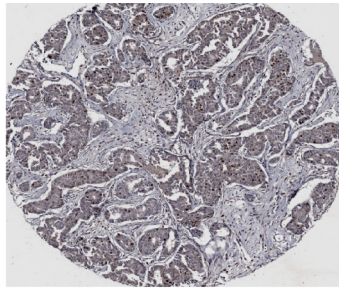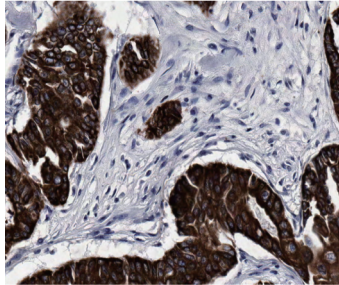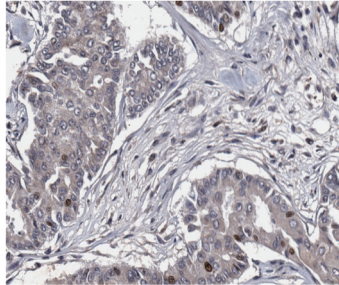

LN (+)

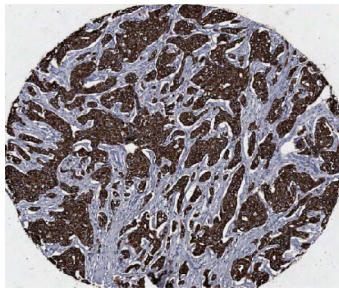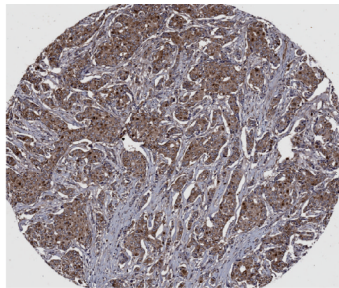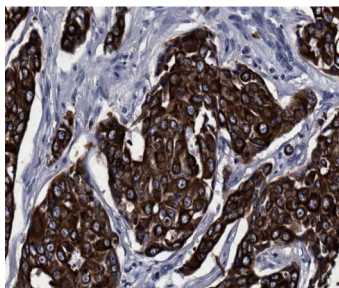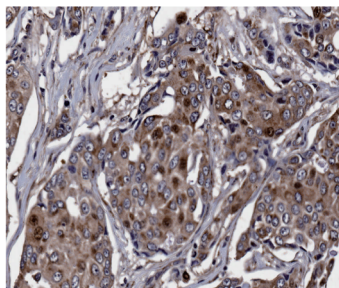

Figure S9

Supplement: Additional file 10 — Figure S9: p21 is specifically overexpressed in breast tumor cells. Representative immunohistochemistry images of AE1/AE3 and p21 in lymph node negative (LN-) and positive (LN+) of breast cancer tissue microarray samples. [file bcr3322-S10.PDF]
